# An approach for the identification of exemplar sites for scaling up targeted field observations of benthic biogeochemistry in heterogeneous environments

**DOI:** 10.1007/s10533-017-0366-1

**Published:** 2017-08-01

**Authors:** C. E. L. Thompson, B. Silburn, M. E. Williams, T. Hull, D. Sivyer, L. O. Amoudry, S. Widdicombe, J. Ingels, G. Carnovale, C. L. McNeill, R. Hale, C. Laguionie Marchais, N. Hicks, H. E. K. Smith, J. K. Klar, J. G. Hiddink, J. Kowalik, V. Kitidis, S. Reynolds, E. M. S. Woodward, K. Tait, W. B. Homoky, S. Kröger, S. Bolam, J. A. Godbold, J. Aldridge, D. J. Mayor, N. M. A. Benoist, B. J. Bett, K. J. Morris, E. R. Parker, H. A. Ruhl, P. J. Statham, M. Solan

**Affiliations:** 1 Ocean and Earth Science, University of Southampton, National Oceanography Centre, Southampton, SO14 3ZH UK; 2 Centre for Environment, Fisheries and Aquaculture Science, Pakefield Road, Lowestoft, NR33 0HT UK; 3 National Oceanography Centre, 6 Brownlow St, Liverpool, L3 5DA UK; 4 Plymouth Marine Laboratory, Prospect Place, The Hoe, Plymouth, PL1 3DH UK; 5 National Oceanography Centre, University of Southampton Waterfront Campus, European Way, Southampton, SO14 3ZH UK; 6 Scottish Association for Marine Science, Scottish Marine Institute, Oban, Argyll, PA37 1QA UK; 7 LEGOS, University of Toulouse, IRDm CNES, CNRS, UPS, 14 av. Edouard Belin, 31400 Toulouse, France; 8 School of Ocean Sciences, Bangor University, Menai Bridge, LL59 5AB UK; 9 Navama – Technology for Nature, Landshuter Allee 8, 80637 Munich, Germany; 10 School of Earth and Environmental Sciences, University of Portsmouth, Burnaby Road, Portsmouth, PO1 3QL UK; 11 Department of Earth Sciences, University of Oxford, South Parks Road, Oxford, OX1 3AN UK; 12 Biological Sciences, University of Southampton, Life Sciences Building, Highfield, Southampton SO17 1BJ UK

**Keywords:** Benthic biogeochemistry, Continental shelf seas, Ecosystem services, Blue carbon, Nutrient cycling

## Abstract

Continental shelf sediments are globally important for biogeochemical activity. Quantification of shelf-scale stocks and fluxes of carbon and nutrients requires the extrapolation of observations made at limited points in space and time. The procedure for selecting exemplar sites to form the basis of this up-scaling is discussed in relation to a UK-funded research programme investigating biogeochemistry in shelf seas. A three-step selection process is proposed in which (1) a target area representative of UK shelf sediment heterogeneity is selected, (2) the target area is assessed for spatial heterogeneity in sediment and habitat type, bed and water column structure and hydrodynamic forcing, and (3) study sites are selected within this target area encompassing the range of spatial heterogeneity required to address key scientific questions regarding shelf scale biogeochemistry, and minimise confounding variables. This led to the selection of four sites within the Celtic Sea that are significantly different in terms of their sediment, bed structure, and macrofaunal, meiofaunal and microbial community structures and diversity, but have minimal variations in water depth, tidal and wave magnitudes and directions, temperature and salinity. They form the basis of a research cruise programme of observation, sampling and experimentation encompassing the spring bloom cycle. Typical variation in key biogeochemical, sediment, biological and hydrodynamic parameters over a pre to post bloom period are presented, with a discussion of anthropogenic influences in the region. This methodology ensures the best likelihood of site-specific work being useful for up-scaling activities, increasing our understanding of benthic biogeochemistry at the UK-shelf scale.

**Electronic supplementary material:**

The online version of this article (doi:10.1007/s10533-017-0366-1) contains supplementary material, which is available to authorized users.

## Introduction

Continental shelf sediments make up less than 9% of the global seafloor, and yet are responsible for the majority of global benthic biogeochemical cycling of organic matter (Jørgensen 1983). Despite their importance, it is still unclear whether sediments act as a source or sink of nutrients and carbon over extensive regions of the shelf (Nedwell et al. 1993), and the processes that lead to changes in the internal pool of dissolved and particulate nutrients and carbon are not fully understood (Hansen and Kristensen 1997; Kristensen and Kostka 2005). A number of key questions need to be addressed in order to determine the importance of the seafloor in moderating biogeochemical cycling and carbon and nutrient stocks, and to reduce the uncertainty associated with predicting the responses of shelf sea systems to natural variability and anthropogenic forcing, including climate change (Viollier et al. 2003; Gruber 2011; Solan et al. in prep). These include: (1) what are the short term (seasonal to annual/interannual) stocks and flows of carbon and nutrients across a gradient of cohesive to non-cohesive sediments? (2) What is the role of shelf sea sediments in long term (decades to centuries) carbon storage? (3) What is the role of macrofaunal invertebrates in mediating benthic biogeochemistry? And, (4) what influence do natural and anthropogenic disturbances have on these processes? Addressing these questions allows us to establish the generalities of how abiotic and biotic interactions will affect carbon and macronutrient exchange in shelf sea systems, and how they are likely to change in the future.

A mismatch between measurements and models made across different temporal and spatial scales limits our understanding of the biogeochemical processes that operate at the shelf scale (Capet et al. 2016). As it is not technically possible to measure many variables at the scale of the shelf system, detailed studies of representative shelf environments that span the full variety of biogeochemical conditions offer an opportunity to gain mechanistic insights important for the validation of modelling efforts (Savchuk 2002). These field studies are often logistically challenging, resulting in limited datasets relative to the intrinsic spatial and temporal variability of the shelf (Cardoso et al. 2010). To allow successful scaling (of both resolution and extent) from these studies to regional scales, interdisciplinary approaches which integrate both local- and macro-scale data are most successful (Queirós et al. 2015; Painting et al. 2013). However, care must be taken to identify the appropriate temporal and spatial scales whilst designing field programmes or when interpreting collected data (Morrisey et al. 1992). Different scales can be important for different variables (e.g. species richness vs. abundance: Archambault and Bourget 1996; emergent behaviour or lag periods: Godbold and Solan 2013), and there may be critical scale thresholds for estimating biogeochemical dynamics (Zhao and Liu 2014) and/or scale-dependent cascades of influence between variables (e.g. Guichard and Bourget 1998) that must be taken into account.

Given these considerations, shelf-wide studies must combine in situ observations and validation studies as well as manipulative laboratory and field experimentation to identify causal relationships. These must all be integrated using a range of modelling approaches which simulate spatio-temporal dependent changes in biogeochemical cycles and allow mapping of ecosystem functioning and services (Edgar et al. 2016). A major challenge in achieving this goal is that continental shelf seas exhibit high natural variability, both spatially (Mellianda et al. 2015; Stephens 2015; Spinelli et al. 2004) and temporally (Reiss and Kröncke 2005). They are highly spatially heterogeneous in sediment coverage, with seafloor permeabilities ranging over seven orders of magnitude (Spinelli et al. 2004), resulting in both diffusive and advective biogeochemical exchanges occurring in close proximity. The end members (sand and mud) of these sediment types are reasonably well defined (Precht and Huettel 2003; Middelburg and Levin 2009) but much less is known about the intermediate mixed sediment types typical of the shelf. This spatial variability is mirrored in the benthos where distinct meio- and macrofaunal assemblages are associated with changes in sediment characteristics, water depth, and/or habitat heterogeneity over a wide range of scales (LaFrance et al. 2014; Heip et al. 1985), although the mobility of these different communities between closely spaced patches must also be considered (Levinton and Kelaher 2004). In terms of temporal variability, shelf sea water columns tend to be vertically mixed in the winter months, but can become seasonally stratified during the summer due to heating and a reduction in wind and wave-induced mixing (Simpson and Sharples 2012). Stratification is often key to the initiation of the spring bloom, and also has the potential to cause recurring periods of anoxia, associated with changes in trace metals, nutrients and organic matter concentrations as well as benthic communities (Stachowitsch 2014). Modelling has shown significant variability in the timing of the onset and breakdown of stratification (Young and Holt 2007), with increasing air temperatures driving a gradual trend to bring the spring bloom earlier (Sharples et al. 2006).

One problem, common in any representation of a complex environment (e.g. Zhang et al. 2004), is that it is not possible to measure all of the key controlling parameters and processes essential to regional assessments of biogeochemical cycling in all possible permutations of the varied benthic habitats found on the shelf, and at all scales. It is paramount that any in situ measurements, observation or experimentation are carried out at locations that represent appropriate exemplar sites for the subsequent scaling up from point observations to the necessary regional predictions. It has been suggested that the assessment of large numbers of small volume samples gives greater precision than smaller numbers of larger samples (and is often more cost effective; e.g. Downing 1989; Underwood 1996), justifying a high-replication, small sample approach; but due to practical limitations this necessitates a limited targeted area (reducing transit and therefore sampling times).

For logistical reasons, one approach is to choose an area that contains suitable representative habitat types within a constrained geographic region. The choice of area is based on a subset of key controlling variables and ensures that sites are representative of typical conditions and cover the range of heterogeneity found on the shelf, while variations in potential confounding variables can be minimised.

It is likewise important to remember that continental shelves are also under significant pressure from anthropogenic activities. Approximately 40% of the world’s population lives within 100 km of the coast, a density more than 3 times the global average (Cohen et al. 1997). Shelf seas provide economic prosperity, as well as a range of essential services to these populations, including food provision, recreation, waste disposal and increasingly energy production. Many of these uses directly affect the benthic environment e.g. fishing using trawls, which accounts for 99.6% of the spatial footprint of human activities on the seabed (Foden et al. 2010), impacts upon the structure and functioning of benthic communities (Kaiser et al. 1998; van Denderen et al. 2015), and the structure and stability of the bed (Schwinghamer et al. 1998). It is not possible to remove the effects of these pressures when investigating shelf-scale processes in situ, so careful consideration must be given to these when findings are interpreted, including the differences between causative and correlated relationships.

Here we present the approach adopted within the NERC and Defra-funded Shelf Seas Biogeochemistry (SSB) programme to choose representative benthic sites on the UK continental shelf. The overarching objectives of the SSB programme were to (i) assess carbon and nutrients cycling and their controls on primary and secondary production in UK and European shelf seas, (ii) to increase our understanding of these processes and their role in wider biogeochemical cycles, and (iii) significantly improve predictive marine biogeochemical and ecosystem models over a range of scales. The approach taken is one of regional-local–regional scaling, which ensures a maintained focus on the wider regional context throughout the project. Such nested sampling designs have been shown to successfully overcome problems associated with spatial scaling (e.g. Morrisey et al. 1992), but are rarely applied at the outset of large multidisciplinary projects.

## Methodology

**Fig. 1:**
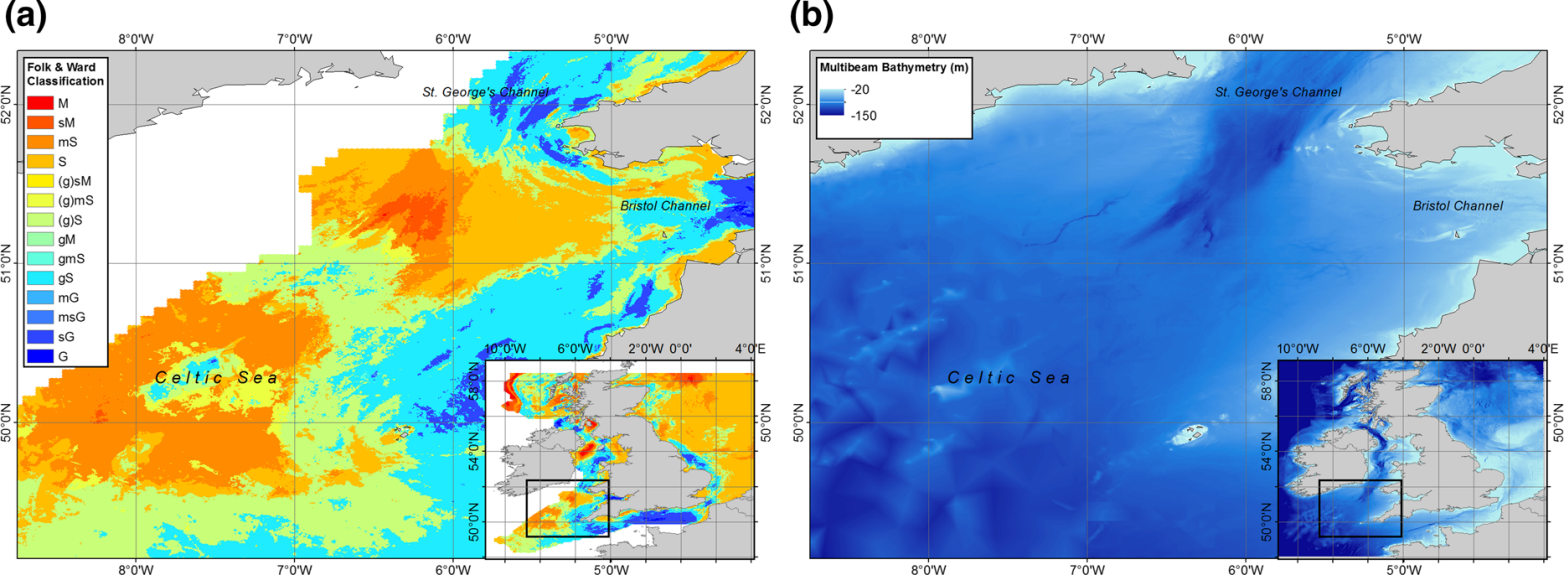
Spatial variations of **a** surface sediment type for the UK shelf (*inset*) and Celtic Sea areas using simplified Folk textural classifications (*M* mud, *S* sand, *G* gravel, with lower case indicating the smaller component of sample, and brackets indicates ‘slightly’; e.g. *mS* muddy sand), based on BGS surface sediment maps (Stephens 2015; Stephens and Diesing 2015; Folk 1954); and **b** Bathymetry, relative to Chart Datum based on 6 arcsec Defra Digital Elevation Map (Astrium 2015)

The Celtic Sea covers an area of approximately 70,000 km^2^ in the Atlantic Ocean to the west of the UK. It exhibits the full range of sediment types typical of the UK shelf, with the additional benefit of varied habitats found in close proximity, and the availability of previous and ongoing monitoring activities in the region (e.g. Davis et al. 2014; Rippeth et al. 2014; Tweedle et al. 2013; Sharples et al. 2013) and over a decade of ecosystem monitoring, research and development funded by the UK government (see “Acknowledgements” for details). It was therefore chosen as an area representative of UK shelf sediment coverage as a whole (Fig. 1a). Comparisons of benthic biodiversity around the UK indicate similarities in infaunal assemblages on both the eastern and western UK shelves, with observed variability dependant on tidal currents and sediment characteristics, and variability in epifaunal assemblages also dependant on sediment type (Rees et al. 1999). This indicates that the Celtic Sea is also a suitable proxy for UK shelf habitats (based on faunal communities; Connor et al. 2004) if variations in sediment type (based on particle size; Folk and Ward 1957) are taken into account.

The site selection procedure involved a three step process in which a constrained target area within the Celtic Sea was chosen, assessed for spatial and temporal heterogeneity, and finally, discrete sites within this area were chosen as suitable for process studies.

### Sampling techniques

The Shelf Sea Biogeochemistry (SSB) programme is an interdisciplinary programme, with wide ranging objectives, aims and deliverables (http://www.uk-ssb.org/). As such, the full methodologies for the techniques used to generate the data presented (including sensor details, calibration methods, method precision and accuracy where relevant) are referenced in the appropriate places within the results section and can be found detailed in Online Resource 1. However, the methods used to collect the samples during an 18 month long cruise programme carried out between 2014 and 2015 are now described. All data collected during the SSB programme is archived with the British Oceanographic Data Centre, (http://www.bodc.ac.uk), and corresponding accession/DOI numbers can be found in Online Resource 1. Unless otherwise specified, statistical relationships between sites are determined using the standard error of the mean, based on the central limit theorem.

### Water column observations and sampling

**Benthic landers** *Continuous Monitoring* A series of benthic landers were designed by the Centre for Environment, Fisheries and Aquaculture Science (Cefas) for continuous monitoring of near-bed water column parameters. They measured conductivity and temperature, pressure, turbidity, oxygen saturation and chlorophyll fluorescence for bursts of 5 min repeated every 30 min at a sampling frequency of 1 Hz. Measurements of currents and backscatter over approximately the bottom 40 m of the water column were recorded in burst mode for 5 min every hour at a sampling frequency of 1 Hz, a temporal resolution sufficient to quantify turbulence. *Intra*-*tidal Monitoring* The National Oceanography Centre (NOC) Liverpool designed the ministable lander to allow shorter-term, higher frequency intra-tidal monitoring of near-bed properties, including velocity, oxygen eddy correlation, water column backscatter (turbidity), bed surface roughness and bedform migration, suspended sediment size, nitrate, temperature, conductivity and depth. **Buoys** Cefas designed *SmartBuoys* provide a long term high-frequency time series (at 1 m below sea surface) of salinity, temperature, turbidity, oxygen saturation, chlorophyll fluorescence, photosynthetically active light climate, and water samples for nutrient analysis. The *M5 Wexford Coast wave buoy* (51.69°N 06.704°W since 2004), part of the Irish Weather Buoy Network provided long-term wave parameters for the region. Lander and Buoy deployment locations and durations can be found in Online Resource 2. **Underway data** pCO_2_ and chlorophyll *a* data were collected while underway throughout the cruise programme. **CTD** Water column profiles of temperature, salinity, depth, chlorophyll fluorescence and turbidity were collected, along with water samples for sensor calibration, nutrient and Iron analysis using both standard and titanium (ultra-clean) Sea-Bird CTD systems. **Fishing Activity** Fishing activities and intensities were assessed using the AIS (Automatic Identification System) and Autosub sidescan imaging.

### Benthic sampling

**Autonomous underwater vehicle survey** The *Autonomous Underwater Vehicles* (*AUVs*) Autosub3 and Autosub6000 (e.g. Morris et al. 2014) were used to survey the study sites using swath bathymetry, sidescan sonar and photography. **Coring** Principal sediment sampling was carried out using a *NIOZ* (*Haja*) *Boxcorer* (*K16*) with 320 mm diameter cylindrical core barrels. In many cases these were then sub-sampled to provide specific sized cores or sediment samples for subsequent experimentation and analysis. Larger sediment samples for faunal analysis were collected using an USNEL-type 500 mm square *Scottish Marine Biological Association* (*SMBA*) *Box Corer*. A Bowers and Conelley *Megacorer* was used to take multiple (up to 12) simultaneous sediment samples for iron pore-water analysis (Barnett et al. 1984; Aquilina et al. 2014; Homoky et al. 2013). **Trawls** A Cefas 2 m *Jennings beam trawl* (Jennings et al. 1999) was used for the collection of epifauna from 3 replicate 5 min trawls carried out a ship speeds of 1.5 knots. **Sediment Profile Imaging** A *Sediment Profile Imaging* (*SPI*) camera was used to capture in situ vertical profile images of the top few centimetres of the seabed, including the sediment–water interface (Rhoads and Cande 1971; Germano et al. 2011).

## Results

### Step 1: Identifying a constrained target area within the Celtic Sea

Given the total area of the Celtic Sea, it was necessary to focus operations on a constrained area that is representative of the Celtic Sea, and the UK Shelf as a whole. The rationale for the selection of this broad target area is based on the identification of varied habitats typical of different sediment types (ranging from fine cohesive muds to coarse advective sands) that exhibit: different biogeochemical exchange mechanisms; varied faunal abundance, diversity and function, while staying within a similar hydrodynamic environment. Confounding variables are reduced by adopting a narrow range of depth, temperature and hydrographic variations. To make this selection, a full assessment of the typical conditions within the Celtic Sea is necessary.

#### Regional hydrodynamics

**Fig. 2:**
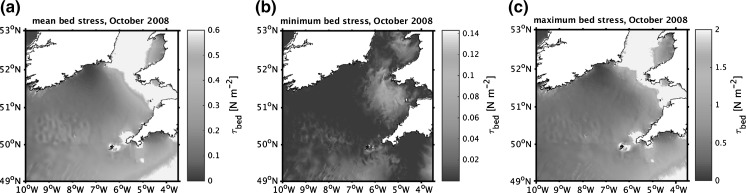
Mean (**a**), minimum (**b**) and maximum (**c**) bed shear stresses (Nm^−2^) typical of winter conditions within the Celtic Sea region. Stresses are obtained from a model simulation for a full year using ~1.8 km resolution for the entire northwest European shelf (Brown et al. 2015) where maximum tidal stresses that year occurred in October

The Celtic Sea extends from the shelf-break at approximately 200 m depth, to a narrow, steep coastal zone. The inner shelf (Fig. 1b) comprises depths between 70–120 m (Uncles and Stephens 2007), and is generally featureless, with a more irregular outer shelf deeper than 120 m. Tides are predominantly semi-diurnal (e.g., Robinson 1979), and the mean spring tidal range increases from approximately 3 m close to its South Western boundary near the shelf break to >12 m in the Upper Severn Estuary in the upper reaches of the Bristol Channel (Hydrographic Office 1996). Spring tidal speeds are relatively low, typically 0.2 m s^−1^ close to the seaward boundary, but increasing to 1.6 m s^−1^ in the Bristol Channel (Uncles and Stephens 2007). Tidal ellipses tend to be strongly elliptical with a clockwise rotation, apart from a localised region of circular ellipses with anticlockwise rotation west of the Bristol Channel (Robinson 1979; Brown et al. 2003; Simpson and Tinker 2009). Tidal ellipses also become more rectilinear as you approach the English Channel. Highly elliptical tidal currents allow for a constantly elevated bed stress, while their polarity influences the height of the bottom boundary layer (e.g. Simpson and Tinker 2009). Bed shear stresses are typically <0.5 Nm^−2^ within the central regions (Fig. 2) increasing towards the shallower English and Bristol Channels to the East and the Irish Sea to the North.

Winds are predominantly from the South West or West, and wave conditions change as the sea becomes shallower and more sheltered. 10-year mean significant wave heights vary from 2 m (8 s peak wave period) near the shelf break to 1 m (6 s peak wave period) where the Celtic Sea meets the Irish Sea, while extreme values for a return period of 1 year reach significant wave heights in excess of 8–10 m and peak periods of approximately 15 s (Bricheno et al. 2015).

#### Water column conditions

Mean winter bottom temperatures are typically 9–10 °C, increasing to 11–16 °C in summer (Uncles and Stephens 2007; Brown et al. 2003). Salinity exceeds 35 near the shelf edge, reducing slightly toward the coast, and varies little seasonally. Winter mixing of the water column in the Celtic Sea leads to a well mixed water column, which is reflected in a homogenous temperature profile between surface and deeper waters. A weak thermocline develops in springtime, which inhibits full water column mixing, providing suitable conditions to initiate a spring bloom (Simpson and Sharples 2012).

Spring blooms in the region are typically dominated by diatoms, which account for up to 80% of primary production during this period (Joint et al. 1986). During the summer months, surface waters become nutrient poor and therefore lacking in phytoplankton. However, the development of a summer deep chlorophyll maximum positioned at the base of the thermocline in the vicinity of the nutricline (Pingree et al. 1977; Hickman et al. 2009) is a well-known phenomenon. Smaller-celled phytoplankton tend to dominate here due to competition for nutrients and include prymnesiophytes, pelagophytes and the cyanobacteria *Synechococcus* (Hickman et al. 2009).

#### Sediment classification

**Fig. 3:**
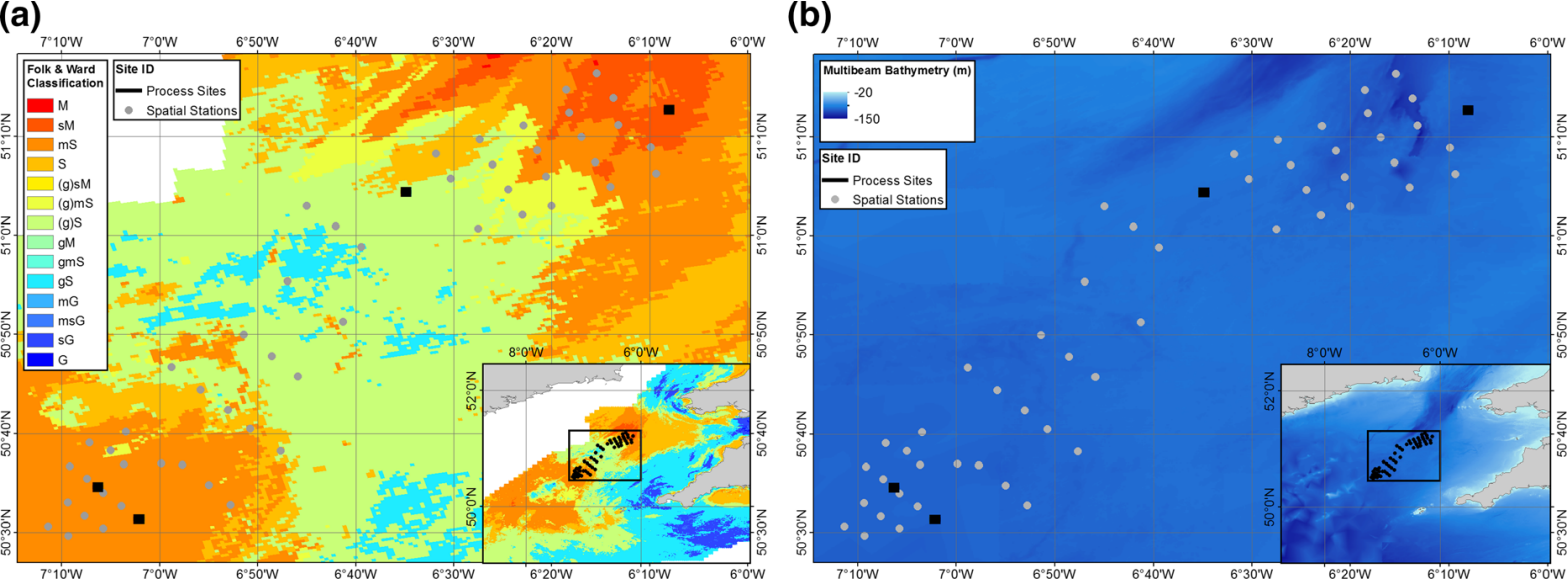
Spatial variations of **a** surface sediment type using simplified Folk textural classifications, based on BGS surface sediment maps (Stephens 2015; Stephens and Diesing 2015; Folk 1954); **b** Bathymetry relative to Chart Datum based on 6 arcsec Defra Digital Elevation Map for the chosen targeted area, overlaid with final sampling station positions (Astrium 2015)

The wider Celtic Sea area contains sediment types ranging from pure muds to gravels (Fig. 1): sediments typical of a shelf-sea environment (bedrock is excluded from the sediment coverage model presented [Stephens and Diesing 2015], however, this has little impact on the project as it’s contribution to biogeochemical cycling is minimal in the UK shelf setting). To ensure a narrow range of depth, temperature and hydrographic variations, a contiguous target area within the inner shelf region of the Celtic Sea was selected with minimal bathymetric variation (Fig. 3b), high hydrodynamic and water column similarity, but also encompassing the widest possible range of seabed types (Fig. 3a).

Within this selected target area, the sediments are dominated by muddy sands, sand, and gravelly sands (comprising 92% of total sediment coverage; Table 1), which typify the wider Celtic Sea region (88% total sediment coverage). The average water depth across the target area is 95 m below chart datum.

**Table 1:** Percentage surface sediment coverage based on Folk Textural Classification categories for the Celtic Sea area in Fig. 1a and the target area in Fig. 4a, highlighting in bold italics those sediment types which comprise >10% of the total (Stephens 2015; Stephens & Diesing 2015; Folk 1954)

Folk classification	Percentage coverage of celtic sea (%)	Percentage coverage of target area (%)
Mud: M	0.005	0.033
sandy Mud: sM	0.838	3.724
muddy Sand: mS	***15.879***	***23.702***
Sand: S	***16.358***	***13.069***
(gravelly) muddy Sand: (g)mS	2.601	4.393
(gravelly) Sand: (g)S	***24.101***	***43.079***
gravelly muddy Sand: gmS	0.150	0.028
gravelly Sand: gS	***31.294***	***11.952***
muddy sandy Gravel: msG	0.165	–
sandy Gravel: sG	8.373	0.020
Gravel: G	0.057	–

#### Fishing activity

**Fig. 4:**
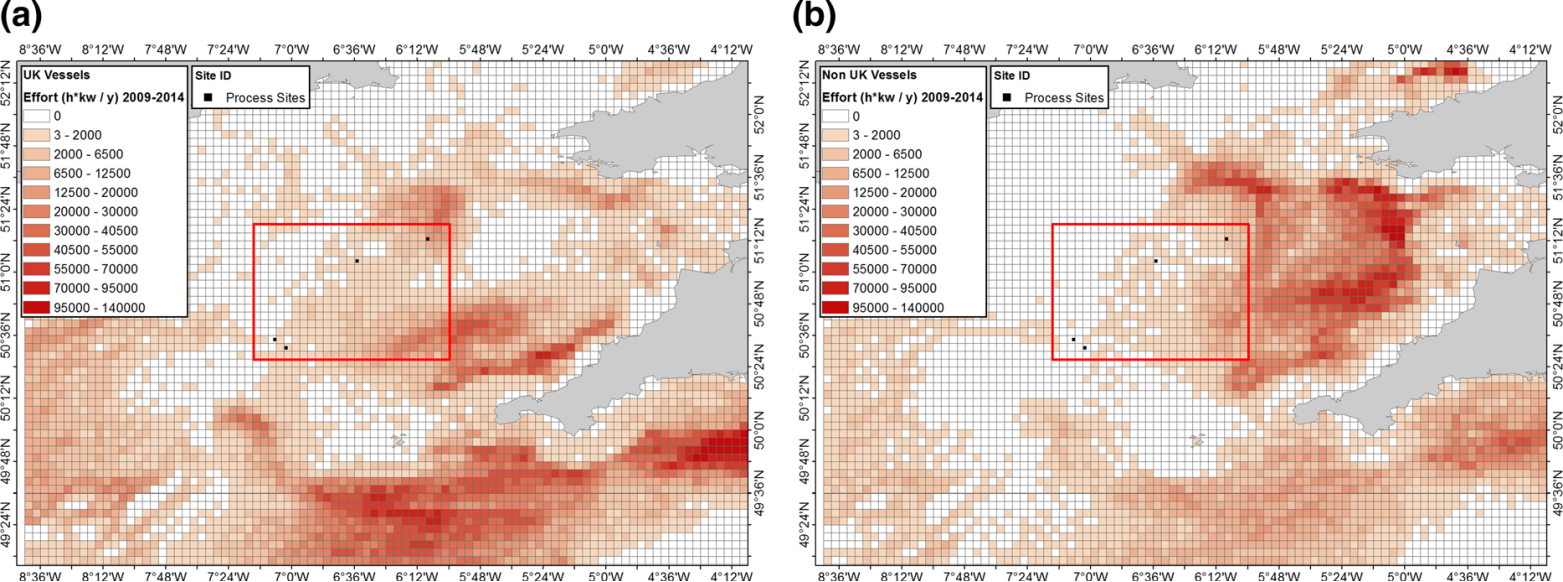
Fishing pressure in the Celtic Sea areas **a** UK vessels and **b** Non-UK vessels. VMS data held by the Marine and Fisheries Agency (MFA) of the UK Department of Environment, Food and Rural Affairs (DEFRA). Calculated effort as Hours times Engine Power per Year (h * kw/y), based on aggregated VMS data of bottom trawled gears, vessel speed between 1–6 knots, from 2009 to 2014. (normalised by year) with cell size 0.05 decimal degrees (following the methods of Lee et al. 2010). Target area (*red box*) and process sites (*black dots*) are identified. (Color figure online)

Large scale commercial fisheries expanded comparatively recently in the Celtic Sea, but have had a relatively large and consistent impact on the area (Blanchard et al. 2005). Fishing activities tend to focus on specific areas (Sharples et al. 2013), targeting the Celtic Deep, shelf edge, and to a lesser extent the central Celtic Sea region (Fig. 4), where trawlers target the Norway lobster *Nephrops norvegicus* on muddy grounds. Fishing occurs year-round at the Celtic Deep (with a slight reduction in Jan-March), although a seasonal pattern is seen in more central regions, with the bulk of activities taking place in spring and summer (Sharples et al. 2013). Vessel Monitoring System (VMS) data from between 2009–2014 suggests a differing trend in fishing ground preferences within the Celtic region when split by UK and non-UK vessels (Fig. 4), likely driven by differences in gear preference, target species, regulations, and fuel prices (Jennings et al. 2012).

### Step 1 summary

The selected target area provides a constrained region on the inner shelf of approximately 87 × 95 km (8265 km^2^) within which to limit long-term observational measurements, cruise operations and in situ experimentation. This restricts sampling to an area of minimal topographic and depth variation, away from the shallower coastal regions where bed stresses are higher, and increasingly varied, and away from freshwater inputs which would affect salinity and temperature. The area contains a wide range of sediment and therefore habitat types, and minimises variations in depth and regional hydrodynamics. To further limit potential depth and hydrodynamic variations, an approximately 20 km wide transect running from the south-west to the north-east across this region (following the tidal flow and predominant wave directions) was identified. The same selection conditions were met, but the required coverage was reduced to an area of approximately 2500 km^2^. The next step was to make a full assessment of the spatial heterogeneity within this new, limited, target area and select discrete sampling sites suitable for repeat seasonal sampling, and representative of the dominant habitat types and biogeochemical exchange mechanisms of the shelf.

### Step 2: assessments of spatial and temporal heterogeneity within the target area and implications for benthic habitats

**Fig. 5:**
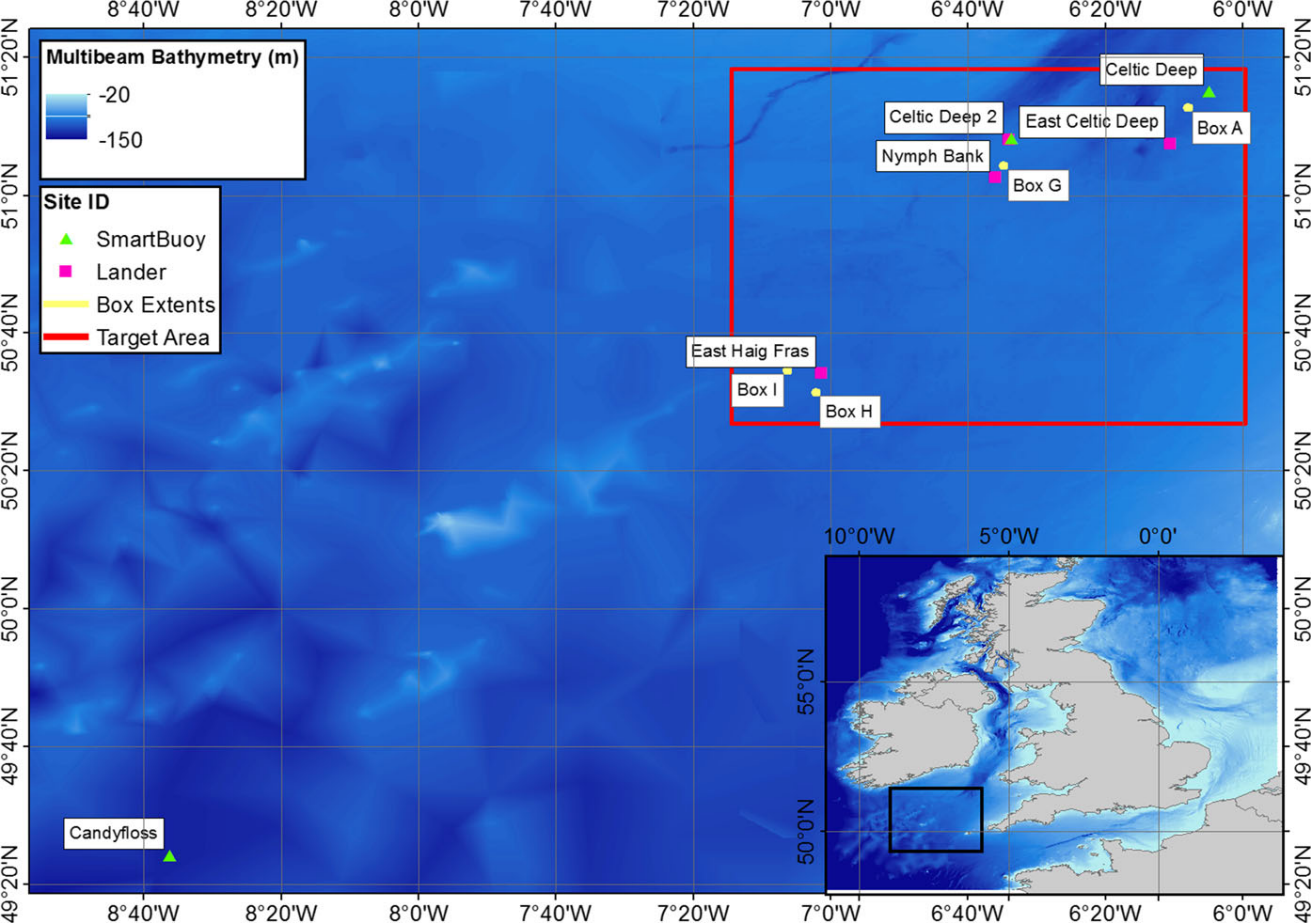
Lander and Smartbuoy positions within the targeted area (outlined in *red*). Locations of the final process study sites also identified. For deployment coordinates, see Online Resource 2. (Color figure online)

The main observational and experimental work for the Shelf Seas Biogeochemistry programme was carried out during 2014–2015. At the start of this cruise programme, a series of benthic landers and SmartBuoys were deployed within the target area to measure long-term hydrodynamic conditions during the survey period (Fig. 5; Online Resource 2).

Four benthic Landers were deployed at The Celtic Deep 2 (CD2L) and East of the Celtic Deep (ECD) both to the North of the region, Nymph Bank (NB) in the central region and East of Haig Fras (EHF) to the South in areas which have similar hydrodynamic regimes (depth, temperature, current direction), but a range of bed types. Consideration was also made to existing infrastructure: a SmartBuoy has been located at the Celtic Deep (CD) site since 2009, and was moved to Celtic Deep 2 (CD2) in 2012. In addition, a SmartBuoy was located at the shelf edge (Candyfloss) for assessments of shelf exchanges and links to the pelagic component of the SSB programme (http://www.uk-ssb.org/science_components/work_package_1/).

#### Regional hydrodynamics

**Fig. 6:**
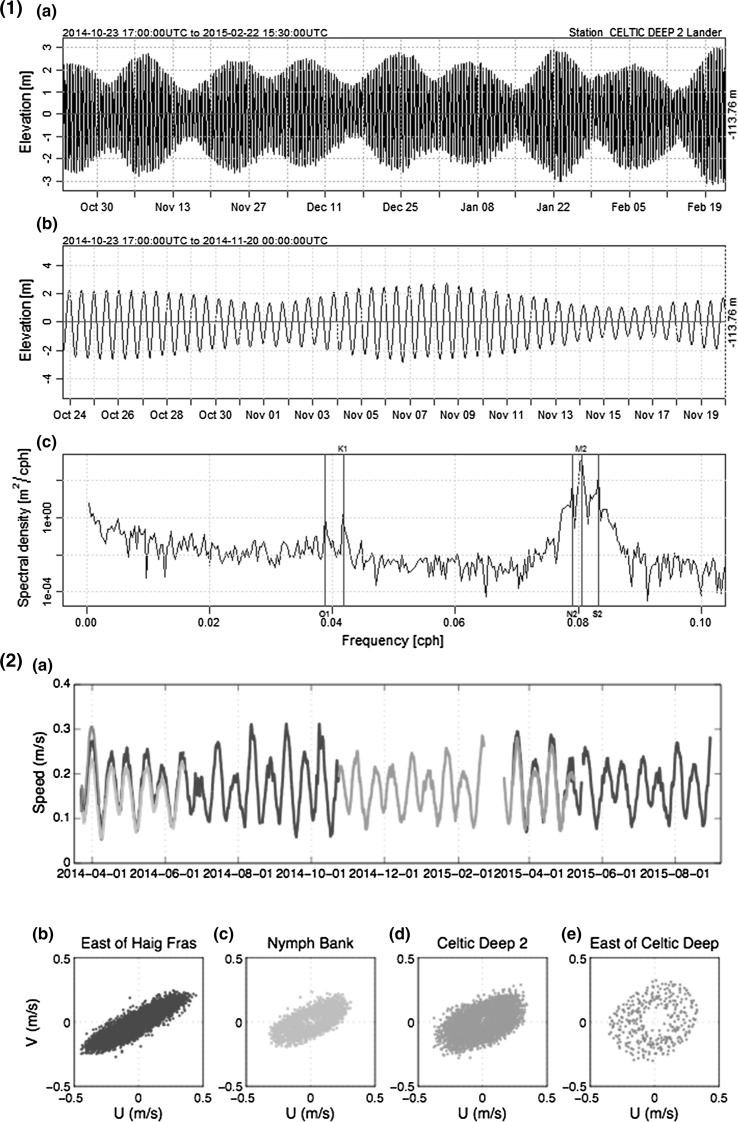
Tidal characteristics at: **1** Celtic Deep 2 Lander site. Showing (*a*) whole deployment elevation, (*b*) first month and (*c*) cumulative spectral density with main tidal components highlighted. **2** The four lander sites. Showing (*a*) 25-h running average of current speed at 2.9 m above the bed (*b*–*e*) Tidal ellipses for the four lander deployments, where U = East and V = North; *colour scheme* maintained between panels (**2** *a*) and (**2** *b*–*e*)

Measured tides in the target area (Fig. 6) are dominated by the M2 tidal constituent, followed by S2 and N2 constituents resulting in semi-diurnal tides with significant spring-neap variations (Robinson 1979). Total spring and neap amplitudes reach 3.1 and 1 m, respectively, at CD2L (Fig. 6a), reducing in the south to 2.9 m springs at EHF, and increasing to the east to 3.4 m springs at ECD consistent with the wider shelf area. Measured near-bed currents are also summarised in Fig. 6(2). While there is little difference in the lowpass current magnitude, the maximum spring currents are strongest at EHF (mean maximum spring current approximately 0.4 m s^−1^), followed by CD2L and ECD (0.36 m s^−1^) and weakest at NB (0.32 m s^−1^). There is a similar behaviour for the maximum bed shear stress (mean spring maximum value of 0.60 Nm^−2^ at ECD, 0.48 Nm^−2^ at ECD and CD2L, and 0.37 Nm^−2^ at NB), but the minimum bed shear stress is significantly higher at ECD (0.02 Nm^−2^ vs. zero at the other three locations) resulting in an increase of the mean bed shear stress. The tidal ellipses also vary from near circular ellipses at ECD to near rectilinear at EHF matching the expected behaviour of the wider Celtic Sea region, with the polarity of the ellipse anti-clockwise for ECD, CD2L and NB, but clockwise for EHF.

Mean daily wind speeds between 2012 and 2015 were 8.1 m s^−1^, with a maximum of 22.9 m s^−1^. There is a strong seasonal signal, with daily mean values of 6.5 m s^−1^ during the summer, and 10.3 m s^−1^ in winter. The M5 Wexford coast wave buoy shows winter waves have a mean height of 2.3 m with a maximum recorded height of 8.1 m in January, and summer mean wave height of 1.4 m.

#### Water column conditions

**Fig. 7:**
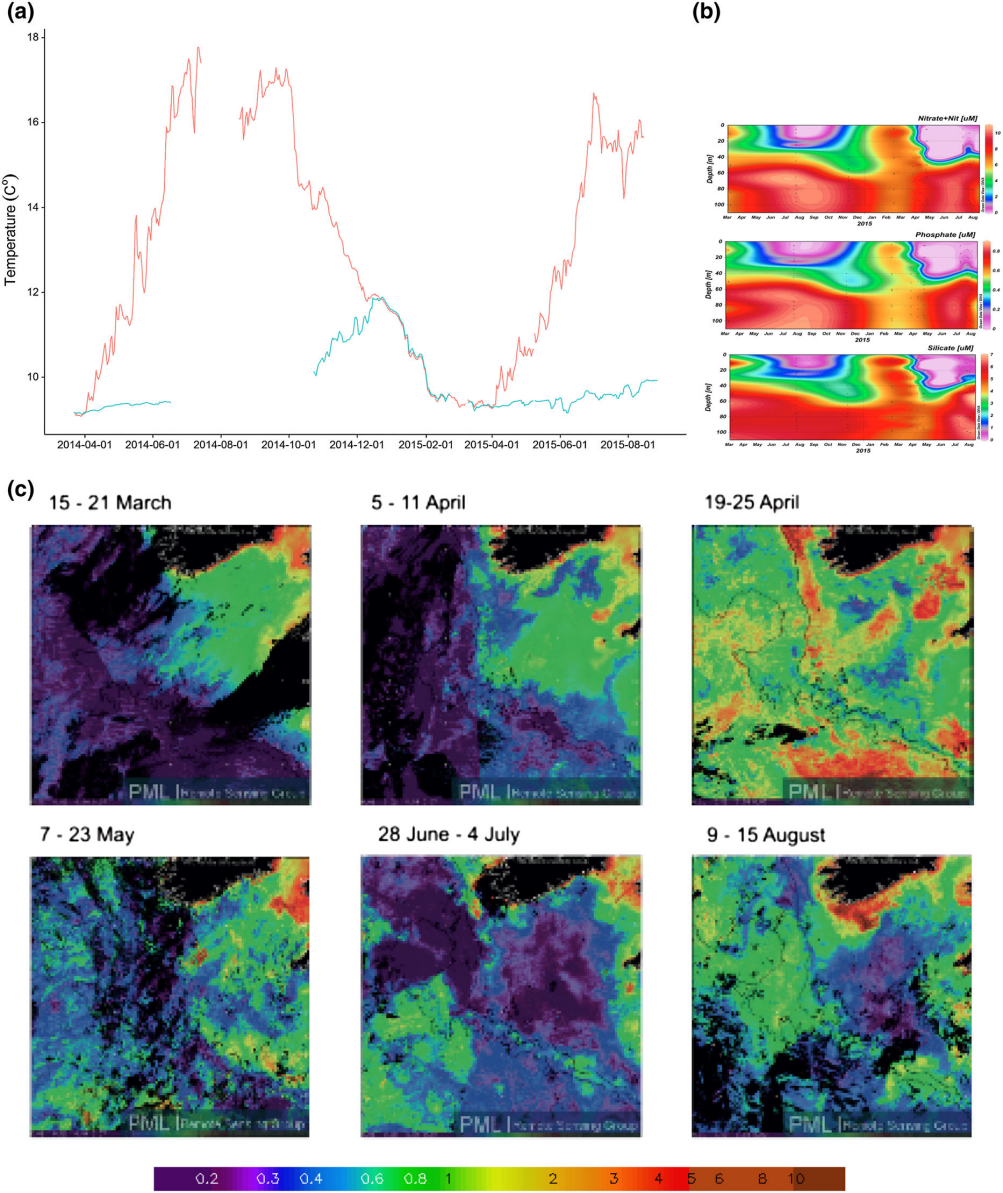
**a** Daily mean temperatures:* red* represents surface temperatures measured by the Celtic Deep 2 SmartBuoy; *cyan* shows near bed temperature measured by the Cefas Continuous Monitoring Lander at Nymph Bank/Celtic Deep 2 Lander sites. **b** Timeseries of nitrate and nitrite, phosphate and silicate (mM) between March 2014 and August 2015 at Celtic Deep. **c** MODIS Surface chlorophyll (mg L^−1^) for the Celtic Sea, March–August 2015. (Color figure online)

Measured surface temperatures since 2009 ranged between 8.06–19.73 °C (mean 13 °C). Stratification formed in early April in both 2014 and 2015, with re-mixing in mid-December in 2014. This is in keeping with prior observations (Brown et al. 2003). CTD data indicate that the mixed layer depth was shallowest in August (~25 m), deepening from September. Surface temperatures during the sampling period were typical of the overall temperature range in the Celtic Sea, with bottom temperatures limited to ~12 °C (Fig. 7a), reaching a maximum following re-mixing during the winter months, and also closely following the trend for the wider Celtic Sea region. Salinity has a narrow range between 34.8 and 35.3 as expected for this inner region of the shelf. Riverine input from the southern coast of Ireland is relatively minor. Freshening during winter and spring is thus primarily attributable to input from the River Severn (Brown et al. 2003). Profiles of PAR allow calculation of vertical attenuation coefficients (Kd; Kirk 2003) between 0.1 and 0.25 m^−1^ in Summer and Autumn, also typical of offshore shelf waters (Foden et al. 2008). Water clarity reaches higher values in summer (ranging from 0.13 and 0.9 m^−1^) and is limited in range in winter (0.2 and 0.4 m^−1^).

The timing of the thermal stratification observed was supported by water column macronutrient profiles collected from CTD deployments over the course of both pelagic and benthic SSB field campaigns (Fig. 7b). During winter months the water column is completely mixed with total oxidised nitrogen (TOxN) concentrations between 6.3 and 6.8 μM at all water depths (March 2015). Similarly, profiles of silicate (range 4.6–5.2 μM) and phosphate (0.56–0.77 μM) demonstrate the homogeneity of the water column at that time. In early April 2015 the onset of stratification and assimilation of nutrients is witnessed with surface concentrations of TOxN depleting to 4.9 μM while bottom water concentrations increased to 7.4 μM. Silicate and phosphate followed suit but depletion was not as pronounced, with surface concentrations at 4.3 and 0.4 μM, and bottom concentrations at 5.1 and 0.6 μM, respectively. By the end of April 2015 once the bloom had successfully established, a strong nutricline is observed between 20 and 30 m. Here, nitrate concentrations have been significantly depleted in surface waters to 0.01 μM, whilst bottom water concentrations have increased further to 10.6 μM. Depletion of surface silicate (0.3 μM) and phosphate (0.01 μM) is also witnessed with elevated concentrations of 5.7 and 0.8 μM, respectively, found at depth. These nutrient conditions are observed throughout the late spring/summer period until the nitrate and phosphate surface water concentrations are further depleted, falling below detection limits (Woodward and Rees 2001). This highlights the biological drawdown of nutrients from the surface waters and probable remineralisation of organic matter at depth, combined with the absence of water column mixing during this period.

Data from SmartBuoys show that phytoplankton blooms are variable in both timing and magnitude in the region, usually occurring in March or April. In 2011, peak Chlorophyll concentrations occurred in March, reaching 16 μg L^−1^. During the SSB survey period, maximum Chlorophyll peaks were lower (3–4 μg L^−1^) and occurred later in the season. Moderate Resolution Imaging Spectroradiometer (MODIS; NASA) satellite data demonstrate that the spring bloom was initiated in early April 2015 coinciding with the onset of stratification, with full bloom conditions observed by mid-April 2015 (Fig. 7c). The bloom lasted for approximately four weeks before crashing by mid-May. During the summer months when surface waters were nutrient poor, the phytoplankton population was reduced.

#### Sediment classification

**Fig. 8:**
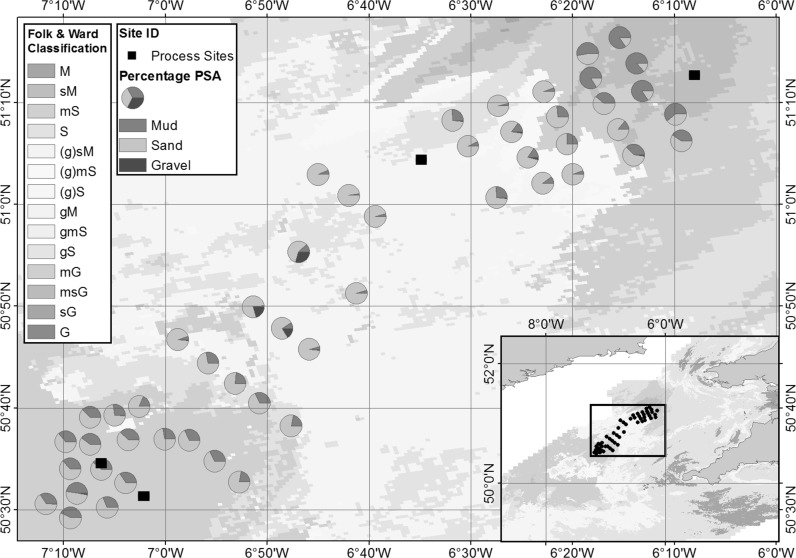
Target area particle size analysis of sediment samples 0–5 cm depth analysed following the NMBAQC method (Mason 2011) overlaid onto interpolated surface sediment map (Stephens 2015; Stephens and Diesing 2015; Folk 1954)

During March 2015, a broad-scale spatial benthic survey was completed to assess the heterogeneity of the sediments within the previously defined target area (Fig. 8). At each sampling location NIOZ box cores were collected and subsampled for particle size, bulk sediment characteristics (bulk density, porosity, permeability and organic content), oxygen and pH profiles, pore-water nutrient concentration profiles and meio- and macro- faunal assessment (see Online Resource 1 for full methodologies). SMBA cores were taken for measurements of megafaunal abundance and assemblage. SPI images were collected for visual determination of sediment type, zone of mixing (previously the apparent redox potential discontinuity [aRPD]; Teal et al. 2010) and bed roughness.

The full results of the survey will be reported in detail elsewhere (e.g. McCelland et al. 2016; Silburn et al. in prep), and confirmed that the targeted area contained a range of sediment types from sandy muds, through to gravelly sands, reflecting the wider shelf region (For full details, see Online Resource 3). In summary, coarser sediments dominate the central region, and the percentage of fine sediments (median grain size < 63 μm), which ranges between 1.73 and 86.61% across the entire area, increases towards the Northeast and Southwest corners (Fig. 8). Multivariate statistical analysis of particle size data suggested that the sites could be allocated to one of eight different seabed types that corresponded well to the Folk and Ward (1957) textural group classifications for sediment bed types. The majority of the samples (92%) were poorly to very-poorly sorted, fine to very-fine skewed (80%) and mesokurtic to very leptokurtic (96%). When overlaid on the targeted area it is clear that the sediment coverage map is successful at representing the range and spatial distribution of surface sediments in the Celtic Sea.

Faunal analysis of the spatial survey samples demonstrated that sediment particle size distributions were generally a good predictor of macrobenthic community structure (McClelland et al. in prep). However, there was considerable overlap in community composition between closely related sediment types. This was due principally to many benthic species present having broad habitat preferences occurring in multiple sediment habitats. In addition, despite changes in community composition between sediment types, levels of macrofaunal abundance, biomass and diversity remained largely constant across all the samples with perhaps only a slight reduction in these parameters for the sites with the highest fines percentages to the Northeast (McClelland et al. 2016). Given that these sites were also subjected to the greatest intensity of trawling, this slight reduction may be due to anthropological disturbance rather than to any natural ecological process.

### Step 2 summary

The spatial survey demonstrated that the target area contains a wide range of benthic sediment and habitat types typical of the wider Celtic Shelf region, while being exposed to minimal variations in water depth, water column conditions and hydrodynamic forcing spatially, which all fall within the ranges expected of the wider Celtic Sea area, but exhibit clear seasonal changes.

### Step 3a: identify and describe exemplar sites; Physical Parameters

Final site selections were made based on the sediment maps and past cruise data presented above, and were further refined using ground-truthing during the first SSB cruise in 2014 (Table 2), and the spatial survey in 2015. Based on the sediment coverage data, four final process sites were selected within the targeted area, which represent the overall range of habitat and sediment types within the region, ranging across the end-member biogeochemical exchange mechanisms (diffusive and advective). Discounting the gravel dominated sediments, due to the practicalities of using the proposed experimental methods on gravels, there are four main sediment types across the target area: mud; sandy mud; muddy sand; and sand. Pure mud is of negligible coverage (0.005%) and so the sites chosen are a sandy mud (with as low a sand fraction as possible) to represent the diffusive end member, a sand sediment to represent the advective end member, and two muddy sand sites in between.

**Table 2:** Sampling and cruise periods, with central points of each 500 m × 500 m process site box

Cruise^a^	Start date	End date	Description
DY008	18 March 2014	13 April 2014	Pre-bloom, site identification and ground truthing
DY021	01 March 2015	26 March 2015	Pre-bloom, spatial survey
DY030	04 May 2015	25 May 2015	Bloom
DY034	06 August 2015	02 September 2015	Post-bloom

Process site name	Benthic A	Benthic I	Benthic H	Benthic G
Central point location	51°12.6754 −6°8.0277	50°34.5557 −7°6.3161	50°31.3329 7°2.142	51°4.3569 −6°34.866

^a^Benthic sampling cruises which took place aboard the RRS Discovery. Where available cruise reports and data inventories can be found at the following link: http://www.uk-ssb.org/research_cruises/programme

Each process site is represented by a 0.25 km^2^ box (500 m × 500 m) within which sampling is constrained, minimising local heterogeneity while ensuring sufficient space to resample the sites without on-going impacts from previous sampling efforts. Process site names represent the order in which they were ground-truthed and are presented according to decreasing fines percentage. The boxes with the highest percentages of fines (A) and sand (G) are used to represent the end-members of the observed spectrum, with the sites H and I displaying intermediate values on the continuum.

The full benthic Shelf Seas Biogeochemistry programme visited each site four times, to assess seasonal differences across each of the sites, and assess conditions prior to, during and after the spring bloom (Table 2). Much of this seasonal data is presented in full within the other contributions to this special issue.

These cruises used a combination of in situ observation, sediment and biological sampling and experimentation to make assessments of biogeochemical processes occurring at each of the sites. While site selection was based on data collected in DY008 and DY021, the data presented below represent typical values averaged over all four cruises, to provide baseline ranges throughout the year for each site, providing the most thorough assessment of site representativeness to the wider target area and Celtic Sea region.

#### Water column conditions

The long-term Lander data can be used to assess the hydrodynamic conditions occurring at the process sites (Table 3), to confirm whether the confounding variables were well constrained. The average water depth of the four sites is 106 m, and between site variation less than 10% of the total average water depth. This is confirmed by Autosub3 collected bathymetry data (Online Resource 4). Bottom temperatures over the sampling period average 9.76 °C, varying within 5% between sites; salinity was 35.2 (<1% variation between sites). Significantly different spatial variations in turbidity (standard error of the mean; p < 0.0001) and O_2_ saturation (p < 0.0001) are apparent which, given the water column similarities between the sites, likely result from differences in the bed sediment or habitat type. Turbidity is highest at ECD, which also corresponds to the highest O_2_ saturation.

**Table 3:** Continuous monitoring lander data

Site	Pressure^a^ (dBar)	Temperature (°C)	Salinity	Turbidity (FTU)	O_2_ saturation (%)
East of celtic deep	104 ± 1.5 (n = 6285) (100–107)	9.56 ± 0.2 (n = 6285) (9.22–10.46)	35.23 ± 0.01 (n = 3200) (35.1–35.27)	9.2 ± 13 (n = 2393) (1.3–178.2)	98.4 ± 3.4 (n = 3200) (91.7–103.9)
Nymph bank	110.5 ± 1.5 (n = 4173) (107.6–113.5)	9.32 ± 0.09 (n = 4173) (9.12–9.46)	35.2 ± 0.0 (n = 4173) (35.13–35.24)	4.3 ± 8.4 (n = 6167) (0.6–89.8)	97 ± 5.3 (n = 4173) (87–104)
East of haig fras	107.5 ± 1.3 (n = 23,702) (104–111.7)	10.13 ± 0.61 (n = 23,704) (9.15–11.81)	35.26 ± 0.05 (n = 12,926) (34.86–35.36)	2.5 ± 4.6 (n = 24,257) (0.4–78)	91 ± 7.0 (n = 10,996) (82–103)
Celtic deep 2 lander	104.2 ± 1.4 (n = 13,975) (94.1–107.4)	10.4 ± 0.8 (n = 13,975) (9.1–11.9)	35.14 ± 0.16 (n = 6407) (34.67–35.36)	2.3 ± 2.2 (n = 14,953) (0.5–65.5)	83 ± 12.9 (n = 6098) (63–106)

^a^Pressure at seabed

**Fig. 9:**
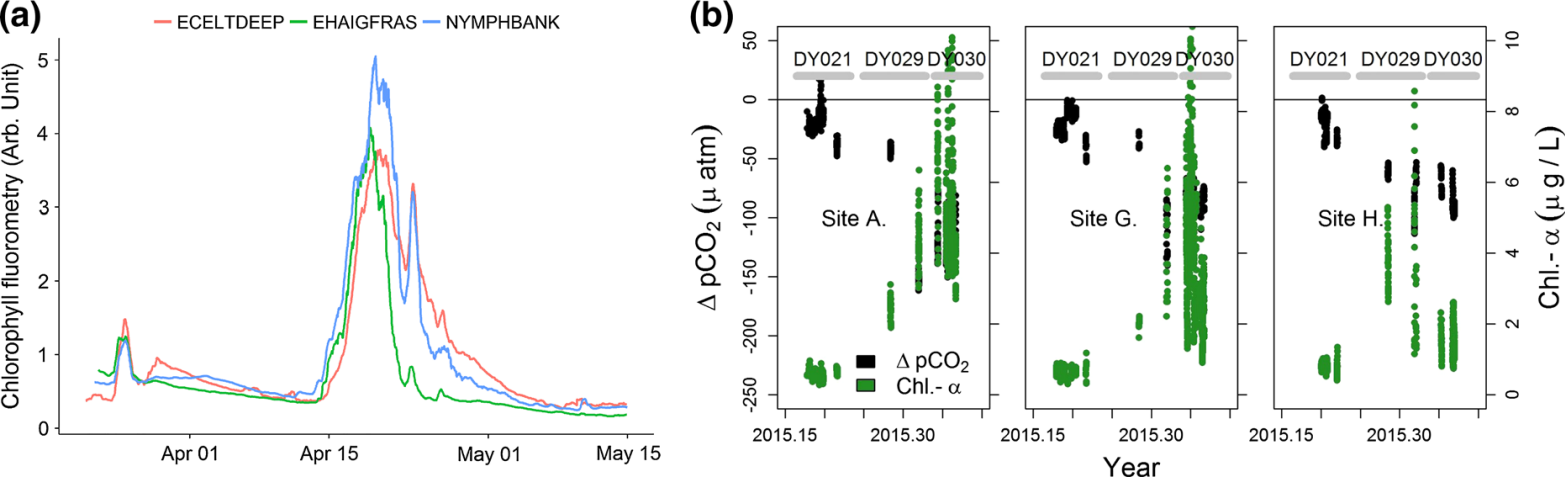
**a** Chlorophyll fluorescence from 2014, indicating concurrent bloom timing. Rolling 24 h mean from Continuous Monitoring Lander. **b** Chlorophyll and sea-air CO_2_ partial pressure gradient (DpCO_2_) at stations A, H and G for 2015

Underway and Lander measured Chlorophyll concentrations indicate that the spring bloom occurred concurrently across the sites, were in agreement with the MODIS satellite data for the Celtic Sea in 2015, and closely correlates with the onset of stratification. The bloom results in similar drawdowns of CO_2_ (Fig. 9b) at each site.

#### Sediment classification

**Fig. 10:**
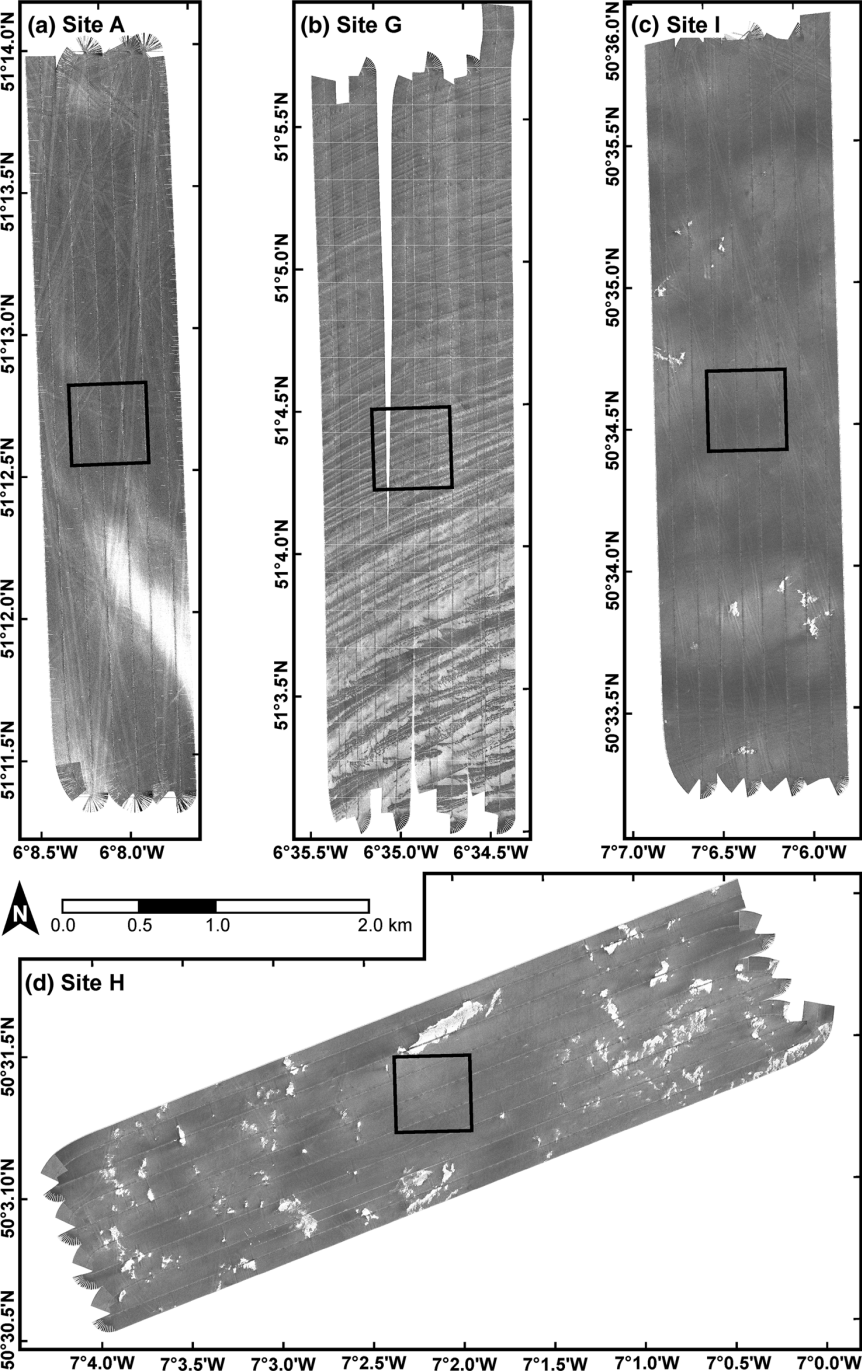
Sidescan surveys of wider areas surrounding the final process site selections. **a** Site A, **b** site G, **c** site I and **d** site H. Close up images from the sites themselves (*black boxes*) can be found in Online Resource 5)

Sidescan surveys were undertaken as part of DY034 using Autosub3 (Fig. 10; Online Resource 5). These encompass the immediate process sites (500 × 500 m black boxes), plus the surrounding areas. High backscatter (light tones) likely represents area of coarser or more mixed sediments, whereas low backscatter (dark tones) finer or more homogeneous sediments. The presence of bedforms at Site G is clear, reducing in wavelength towards the north of the region (from ~130 to ~25 m). These also appear in the bathymetry data collected at site G (Online Resource 4). Presumed ‘trawl marks’ are particularly evident at Site A, but also present at sites I and H.

**Fig. 11:**
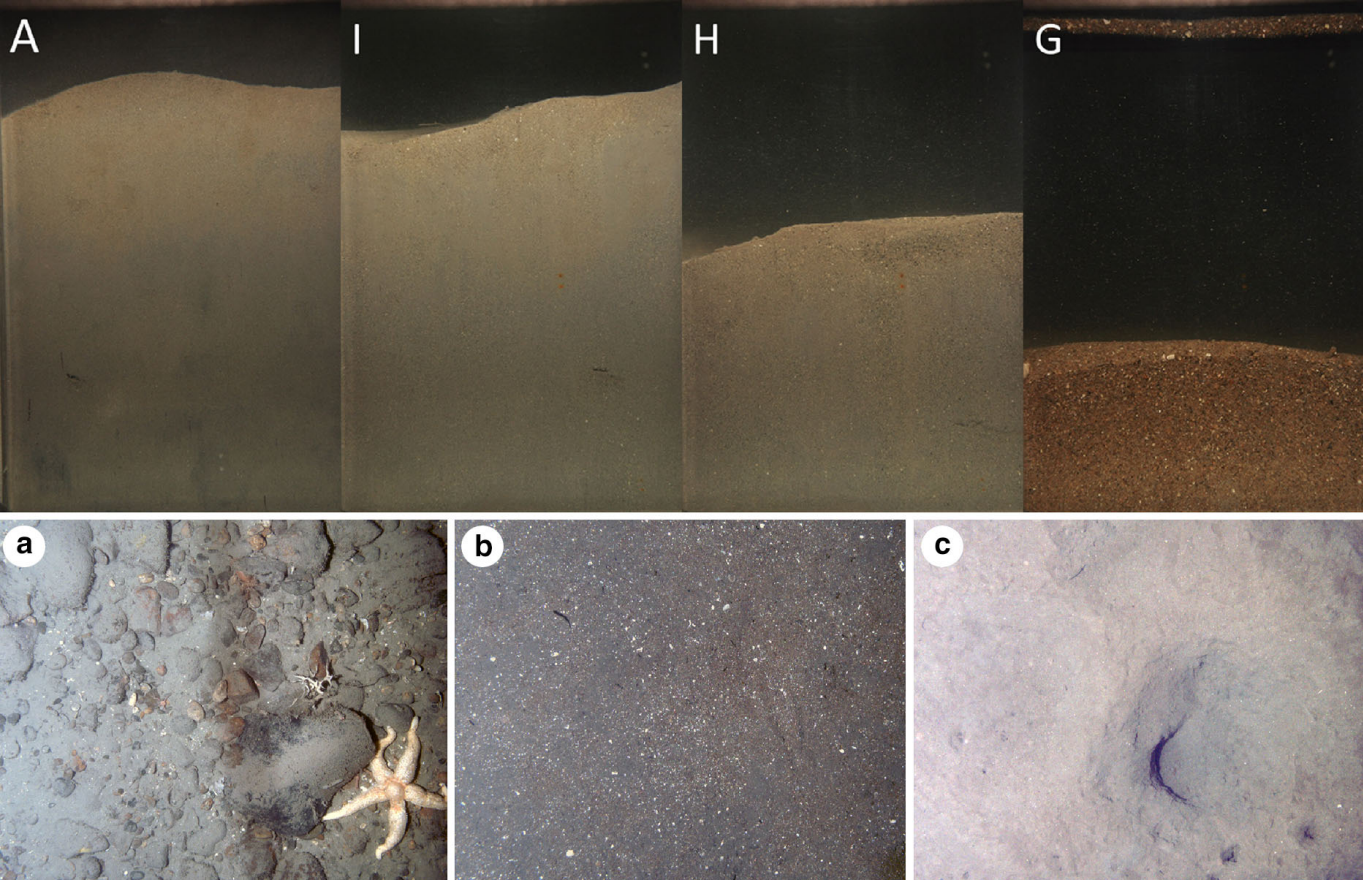
(*Top*) Sediment Profile Imagery (SPI) of the sediment–water interface and sub-surficial sediment profile at the 4 process sites. Image width 15 cm. (*Bottom*) Autosub3 images of **a** hard; **b** intermediate and **c** soft sediment types

SPI images (Fig. 11) from the four process sites show clear visual differences in grain size, surface roughness and sediment colour indicative of different sediment and habitat types. Photographs from the Autosub3 survey were used to visually distinguish between habitat types and could be divided into three broad categories: hard (Fig. 11a: >50% of the photograph covered by cobbles or boulders); intermediate (Fig. 11b: 1–49% coverage of granules, cobbles or boulders); and soft (Fig. 11c: 100% coverage by sand or mud). Particle Size Analysis (PSA) of multiple sediment samples taken from NIOZ box cores over the 4 cruises (Table 4) confirms that the differences between mean values at each site are statistically significant.

**Table 4:** Sediment characterization and structural parameters for the four process study sites

Site	Median grain size (d_50_, m)	Mean grain size^a^	Sorting^a^	Skewness^a^	Kurtosis^a^	% fines^b^	Folk class^c^	Dry bulk density (kg m^−3^)	Porosity	Specific permeability^d^ (×10^−14^ m^2^)
Benthic A	57.30 ± 25.70 (n = 20) (15.69–145.66)	37.64 ± 18.5 (n = 20) (15.71–108.24)	4.78 ± 0.52 (n = 20) (4.13-6.18)	−0.45 ± 0.10 (n = 20) (−0.53 to 0.08)	1.00 ± 0.13 (n = 20) (0.68–1.39)	53.65 ± 10.76 (n = 20) (24.04–72.89)	Sandy mud	835.57 ± 142.27 (n = 12) (735.45–1041.11)	0.68 ± 0.05 (n = 12) (0.61–0.72)	2.16 ± 2.10 (n = 12) (0.59–5.25)
Benthic I	121.51 ± 30.33 (n = 20) (51.88–197.52)	88.62 ± 35.13 (n = 20) (33.63–177.69)	4.56 ± 0.83 (n = 20) (3.63–6.37)	−0.40 ± 0.10 (n = 20) (−0.61 to 0.25)	1.36 ± 0.22 (n = 20) (0.94–1.69)	28.36 ± 8.01 (n = 20) (17.10–53.16)	Muddy sand	1119.43 ± 137.98 (n = 12) (983.03–1247.11)	0.58 ± 0.05 (n = 12) (0.53–0.63)	15.4 ± 6.53 (n = 12) (9.12–23.3)
Benthic H	177.63 ± 97.96 (n = 22) (79.48–518.22)	145.67 ± 104.33 (n = 22) (37.05–509.77)	4.19 ± 1.16 (n = 22) (1.88–6.43)	−0.37 ± 0.11 (n = 22) (−0.63 to 0.11)	1.41 ± 0.26 (n = 22) (0.82–1.87)	21.92 ± 8.93 (n = 22) (4.88–43.82)	Muddy sand	1182.19 ± 61.09 (n = 12) (1121.40–1261.08)	0.55 ± 0.02 (n = 12) (0.52–0.58)	57.4 ± 46.6 (n = 12) (25.6–125.4)
Benthic G	458.83 ± 175.14 (n = 20) (48.26–730.33)	445.95 ± 188.75 (n = 20) (29.35–715.82)	3.05 ± 1.9 (n = 20) (1.65–9.58)	−0.30 ± 0.24 (n = 20) (−0.66 to 0.36)	2.17 ± 0.89 (n = 20) (0.48–3.20)	13.05 ± 16.69 (n = 20) (1.98–56.28)	Sand	1493.07 ± 178.36 (n = 12) (1299.84–1714.14)	0.44 ± 0.07 (n = 12) (0.35–0.51)	693.6 ± 180.1 (n = 12) (491.7–857.4)

Values are means of all samples collected at the sites, ±SD (min–max ranges in brackets) and represent bulk samples 0–5 cm in depth

^a^Folk and Ward (1957) geometric (modified) graphical (μm) measures

^b^Fines <63 μm

^c^Folk (1954) textural class

^d^Engelund (1953)

The four sites exhibit statistically different averaged median grain sizes (standard error of the mean; p < 0.005), although H and I fall into the same textural classification (Table 4). In summary: site A is a very poorly sorted, very fine skewed, mesokurtic, very coarse silt, classified according to the Folk classification scheme as a sandy mud; site I is a very poorly sorted, very fine skewed, leptokurtic very fine sand, classified as a muddy sand; site H is a very poorly sorted, very fine skewed, leptokurtic fine sand, also classified as a muddy sand; and, site G is a poorly sorted, fine-very fine skewed, very leptokurtic medium sand.

The structure of the near-bed sediment (top 5 cm) was also assessed for each of the sites (Table 4). Depth averaged dry bulk densities are statistically different between sites (p < 0.005), with the exception of H and I (p = 0.48). Porosity and permeability are significantly different in all cases (p < 0.020 and p < 0.001 respectively). As expected, bulk density and specific permeability both increase with median grain size, while porosity decreases.

**Fig. 12:**
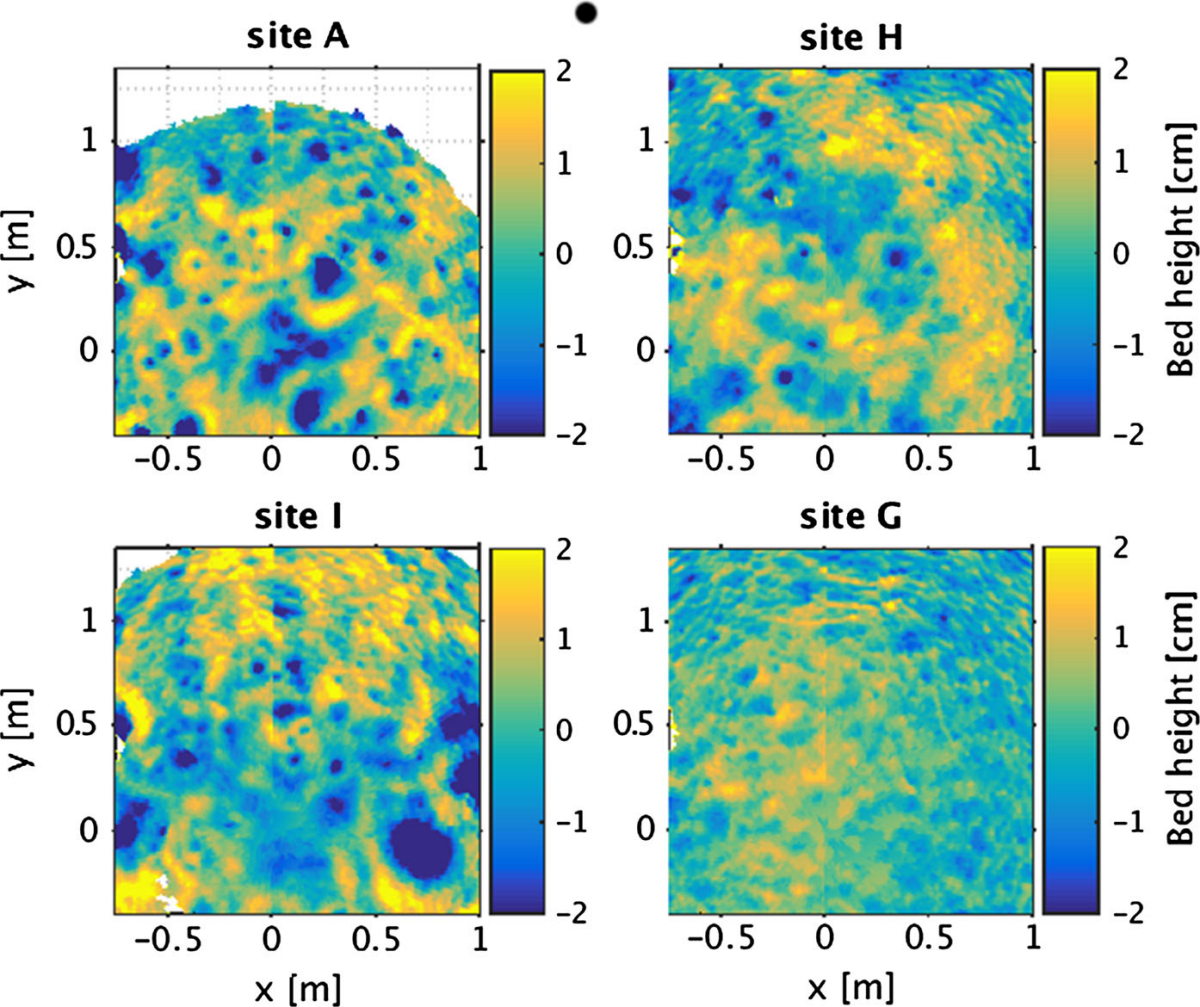
Acoustic images of relative bed roughness from the intra-tidal miniSTABLE Lander, August 2015

Small-scale seabed topography is provided from acoustic images of the bed measured by the 3D Acoustic Ripple Profiler (ARP) on the miniSTABLE intra-tidal monitoring lander. Results for the four sites show a variation in bed height of up to 4 cm (Fig. 12). Bed structures at the more cohesive sites (A, H and I) appear to be dominated by circular depressions, probably caused by benthic fauna. Ripples are observed at the sandy site with little if any migration in all cases. These ripples are predominantly two-dimensional in March and May with ripple height approximately 2–3 cm and ripple wavelength approximately 20-30 cm, and three-dimensional in August with height approximately 1 cm and wavelength approximately 15 cm. The footprint of the ARP is too small to capture the larger scale (~30 m) bedforms seen in the sidescan data. Surface roughness (measured from SPI images; e.g. Fig. 11) is similar at all the muddy sites, and only significantly different at G (p < 0.05), as confirmed from the acoustic bed roughness measurements presented above (Fig. 12).

### Step 3a summary

The analysis described confirms that the four process sites can be considered as statistically different from each other in terms of the sedimentary characteristics (a key scientific variable of the SSB programme), showing a clear and concurrently occurring seasonal signal (key variable), while being similar in terms of hydrodynamic parameters (confounding variables).

### Step 3b: identify and describe exemplar sites; biological and biogeochemical parameters

Assessments were made of key biogeochemical and biological parameters (Tables 5, 6), measured over all four cruises, providing typical ranges found at each site.

**Table 5:** Biogeochemical Parameters

Site	Bulk properties^a^						
	Organic carbon (%)^d^	Organic nitrogen (%)^d^	Oxygen penetration depth (cm)^e^	Total oxygen consumption (mmol^−2^ d^−1^)^f^	Chlorophyll (µg g^−1^)^g^	Zone of mixing (cm)^h^	Surface roughness (cm)^h^
Benthic A	1.12 ± 0.13 (0.98–1.34)	0.13 ± 0.02 (0.10–0.17)	0.875 ± 0.54 (0.3–1.6)	−6.60 ± 3.82 (n = 30) (−13.46 to 9.80)	1.43 ± 0.60 (0.68–2.1)	6.28 ± 0.98 (3.69–7.30)	1.85 ± 0.79 (0.92–3.20)
Benthic I	0.58 ± 0.15 (0.39–0.84)	0.09 ± 0.03 (0.04–0.14)	0.725 ± 0.60 (0.20–1.50)	−7.70 ± 3.97 (n = 24) (−19.13 to 3.17)	0.47 ± 0.17 (0.22–0.6)	5.23 ± 1.54 (3.32–8.01)	1.83 ± 0.53 (1.20–3.30)
Benthic H	0.42 ± 0.12 (0.31–0.65)	0.07 ± 0.02 (0.04–0.11)	0.875 ± 0.49 (0.3–1.5)	−7.97 ± 2.64 (n = 30) (−13.62 to 2.39)	0.42 ± 0.18 (0.3–0.64)	4.55 ± 1.27 (3.05–7.47)	1.82 ± 0.35 (1.25–2.46)
Benthic G	0.22 ± 0.18 (0.11–0.49)	0.06 ± 0.04 (0.02–0.12)	2.08 ± 2.00 (0.5–5)	−4.16 ± 2.82 (n = 28) (−11.02 to 0.66)	0.33 ± 0.26 (0.08–0.62)	n/a	1.50 ± 0.89 (0.61–4.50)

Site	Porewater concentrations (µM)^b^						
	Nitrite (NO_2_^−^)	TOxN (NO_2_^−^ + NO_3_^−^)	Ammonium (NH_4_^+^)	Silicate (SIO_4_^−^)	Phosphate (PO_4_^3−^)	Iron (Fe)^i^	Iron (Fe(II))^i^
Benthic A	0.46 ± 0.37 (0.07–8.27)	4.45 ± 3.60 (0.33–12.8)	38.3 ± 30.1 (0.29–144)	134 ± 83.8 (5.72–339)	7.61 ± 5.94 (0.93–28.4)	4 ± 6 (0.1–10)	3 ± 5 (0.08–9)
Benthic I	0.58 ± 0.75 (0.10–3.14)	4.26 ± 2.97 (0.20–17.9)	38.3 ± 30.9 (0.23–145)	146 ± 88.5 (3.14–358)	7.55 ± 5.98 (0.25–29.0)	7 ± 7 (3–15)	12 ± 15 (3–29)
Benthic H	0.78 ± 1.52 (0.09–1.74)	4.63 ± 4.00 (0.08–9.2)	38.6 ± 27.8 (0.29–107)	145 ± 82.0 (5.39–347)	7.68 ± 5.70 (0.86–25.7)	0.5 ± 0.7 (0.11–1.3)	0.5 ± 0.7 (0.1–1.3)
Benthic G	0.74 ± 1.19 (0.09–2.87)	4.47 ± 3.56 (0.19–16.6)	40.3 ± 30.3 (0.55–114)	153 ± 88.1 (5.93–368)	7.47 ± 5.55 (1.08–21.2)	n/a	n/a

Site	Diffusive fluxes (mmol.m^−2^.d^−1^)^c^					
	Nitrite (NO_2_^−^)	TOxN (NO_2_^−^ + NO_3_^−^)	Ammonium (NH_4_^+^)	Silicate (SIO_4_^−^)	Phosphate (PO_4_^3−^)	Iron (Fe(II))^j^ ×10^−3^
Benthic A	0.013 ± 0.031 (−0.017 to 0.098)	0.019 ± 0.174 (−0.212 to 0.499)	0.021 ± 0.156 (−0.286 to 0.483)	1.212 ± 0.679 (0.206 to 3.741)	−0.018 ± 0.024 (−0.063 to 0.028)	14.4 ± 19.7 (−0.01 to 54.4)
Benthic I	0.012 ± 0.021 (−0.007 to 0.064)	0.125 ± 0.267 (−0.286 to 0.644)	−0.003 ± 0.145 (−0.077 to 0.380)	0.646 ± 0.430 (−0.049 to 1.550)	0.001 ± 0.029 (−0.080 to 0.054)	8.30 ± 10.3 (0.23 to 32.8)
Benthic H	0.011 ± 0.038 (−0.035 to 0.132)	0.082 ± 0.286 (−0.586 to 0.649)	0.049 ± 0.191 (−0.215 to 0.699)	0.702 ± 0.612 (−0.287 to 2.016)	0.004 ± 0.028 (−0.073 to 0.086)	2.7 ± 5.47 (0.06 to 16.8)
Benthic G	0.024 ± 0.030 (−0.008 to 0.105)	0.059 ± 0.133 (−0.131 to 0.599)	0.044 ± 0.023 (−0.257 to 0.319)	0.531 ± 0.474 (−0.007 to 2.255)	0.009 ± 0.035 (−0.085 to 0.070)	n/a

Values are means of all samples collected at the sites, ± standard deviations (min–max ranges in brackets) and represent: ^a^ samples 0-5 cm in depth; ^b^ samples collected seasonally at the sites from triplicate porewater profiles (n = 9) representing 0–10 cm depth; ^c^fluxes calculated at each site (n = 5–11) ± SD (min–max ranges in brackets). ^d^ Kirsten (1979), ^e^Measured immediately from 20 cm diameter cores, sub-sampled from NIOZ box cores, Cai and Sayles (1996); ^f^ Glud (2008), Hicks et al. (2017), Smith et al. (in prep); ^g^ Measured using spectrophotometry (for DY008, HMSO (1980)) or fluorescence (Tett 1987); ^h^ Derived from SPI, Solan et al. (2004); ^i^ Iron values are for surface (0–2 cm) only: not measured at Benthic G; ^j^ Homoky et al. (2012)

**Table 6:** Biological parameters

Site	Epifauna			Macro-infauna (>1 mm)			Meifauna
	Abundance (ind.m^−2^)	Blotted wet weight biomass (g.m^−2^)	Diversity (species)	Abundance (ind.m^−2^)	Blotted wet weight biomass (g.m^−2^)	Diversity (species)	Abundance (k = 1000 × ind m^−2^)
Benthic A	0.88 ± 0.56	2.29 ± 1.65	54	957 ± 603	35.7 ± 82.7	21.2 ± 4.8	806 k ± 281 k
Benthic I	0.9 ± 1.02	0.75 ± 0.23	78	1190 ± 816	10.2 ± 21.4	31.2 ± 10.6	556 k ± 242 k
Benthic H	0.8 ± 0.7	0.57 ± 0.34	128	1130 ± 521	14.0 ± 1.4	37.6 ± 8.1	596 k ± 222 k
Benthic G	1.57 ± 1.61	1.82 ± 0.88	115	483 ± 291	16.0 ± 23.0	21.1 ± 9.1	560 k ± 178 k

Site	Meifauna		Microbes	Bioturbation metrics (mm)				
	Calculated wet weight biomass (g.m^−2^)^a^	Diversity (phyla)	% archael 16S rRNA genes	BPc	^f-SPI^L_max_	^f-SPI^L_mean_	^f-SPI^L_med_	SBR
Benthic A	1.13 ± 0.35	5.7 ± 1.3	29.7 ± 16.5	36.70 ± 22.53	13.12 ± 6.67	4.24 ± 1.70	4.11 ± 1.62	16.27 ± 11.27
Benthic I	1.14 ± 0.48	6.4 ± 2.0	35.8 ± 15.9	19.11 ± 13.14	11.62 ± 4.84	4.35 ± 1.56	4.22 ± 1.49	15.10 ± 7.85
Benthic H	0.73 ± 0.39	4.8 ± 1.2	38.3 ± 20.9	30.31 ± 20.33	15.09 ± 12.32	4.17 ± 1.32	4.08 ± 1.33	14.14 ± 8.80
Benthic G	0.68 ± 0.17	5.9 ± 2.0	22.2 ± 14.2	25.01 ± 17.70	10.03 ± 4.52	4.37 ± 1.64	4.30 ± 1.61	14.69 ± 9.37

Site	Megafauna		Demersal fish		Invertebrates	
	Density (ind m^2^)	Biomass (gm^−2^)	Density (ind m^2^)	Biomass (gm^−2^)	Density (ind m^2^)	Biomass (gm^−2^)
Seabed Photography						
Benthic I	0.53 (0.48–0.59)	6.43 (6.26–6.61)	0.09 (0.07–0.11)	5.21 (5.05–5.41)	0.40 (0.35–0.44)	1.04 (1.03–1.05)
Benthic H	0.59 (0.53–0.65)	14.5 (13.6–15.5)	0.06 (0.05–0.07)	8.75 (8.05–9.50)	0.48 (0.43–0.54)	2.60 (2.52–2.68)
Benthic G	0.57 (0.51–0.63)	4.77 (4.65–4.90)	0.08 (0.07–0.10)	2.54 (2.43–2.64)	0.44 (0.40–0.49)	2.45 (2.37–2.53)

Discussion of specific species abundance can be found in Online Reference 7

^a^ Based on nematodes

#### Biogeochemical parameters

Sediment total organic carbon and total organic nitrogen content are both highest at site A, intermediate at H and I, and lowest at site G. These differences are significant (standard error of the mean; p < 0.05) in all cases, except for organic nitrogen between H and G. These trends are maintained with similar magnitudes when considered seasonally, except for site G, where the core used for analysis had a much higher fines content than typical for this site. Oxygen penetration depths are significantly different only between I and G, although total oxygen consumption rates are significantly different in all cases except between I and H. It should be noted, however, that total oxygen consumption rates are calculated based on the combination of data from three different analytical methods providing total oxygen uptake rates, diffusive oxygen uptake rates and oxygen penetration depths, and are discussed in more detail in Hicks et al. (2017) and Smith et al. (in prep). There are both site and seasonal differences, with more noticeable changes in the cohesive sites, and greatest O_2_ consumption nearest the spring bloom. These seasonal signals are discussed further in Hicks et al. (2017). Chlorophyll measured in the surface sediments at A is significantly higher than the other three sites (p < 0.001), and significantly lower at G than at I (p < 0.05). The zone of mixing is significantly different at all sites (p < 0.05) being lowest at H, and highest at A.

#### Pore waters

**Fig. 13:**
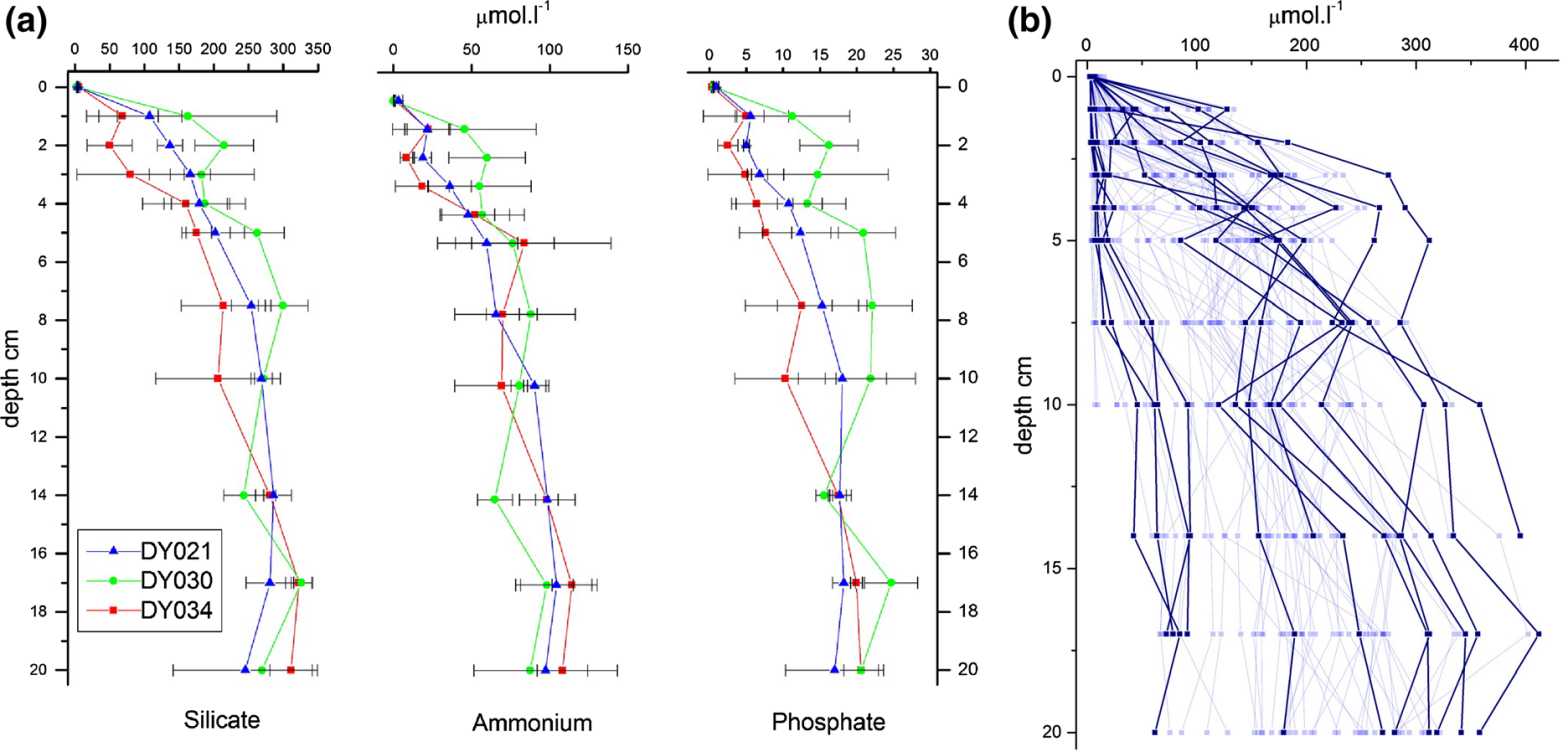
**a** Example pore water profiles with depth, Box I. **b** Example pore water silicate concentrations—main stations (A, I, H, G) in triplicate (*dark blue lines*) overlaid on spatial survey stations collected in March 2015 (*light blue*). (Color figure online)

Pore water nutrient concentrations were measured in triplicate usually down to 20 cm using a depth variable resolution. Data averaged for 0–10 cm are presented (Table 5). The concentration of NH_4_^+^ ranges between 0.23 and 145 μM across all sites and cruises. The concentrations at Sites A, H and I generally increase from the sediment surface to 10 cm depth, and are relatively stable below 10 cm (Fig. 13). At Site G, increases do not occur until below 3–4 cm depth. Silicate profiles show similar trends as the NH_4_^+^ with higher concentrations (3–368 μM).

TOxN is usually at a maximum in the top 2 cm except at Site G where values at depth are occasionally higher than at the surface, with a maximum value of 16.6 μM. Nitrite ranged between 0.07 and 8.27 μM and is generally evenly distributed throughout the top 20 cm. The differences between sites are not statistically significant, however, this is likely due in part to large ranges resulting from measurements averaged over the different seasons (e.g. Fig. 13a). Ranges were similar to those measured over the spatial survey described in step 2 above (Fig. 13b) and therefore considered representative of the region as a whole, and the inherent variability in the profile shapes, likely due to high variability in the vertical sediment structure, should be noted.

Typically, porewater Fe concentration maxima occur in the shallow subsurface (up to >100 μM at approx. 5 cm depth) and decrease sharply across the oxic surface layer (profiles not shown, see Klar et al. this issue). Average surface (0–2 cm depth) porewater Fe concentrations are highest at site I, lowest at site H and intermediate at site A (Table 5). Most of the porewater Fe is in its reduced and soluble Fe(II) form, and our data suggests that oxygen penetration depths (which can be related to e.g. advective transport, bioirrigation or bioturbation) exert a strong influence on pore water Fe contents across the study sites (Klar et al. this issue). Seasonal variations are discussed in detail in Klar et al. (this issue).

#### Diffusive nutrient fluxes

Ten centimetre diameter sediment sub-cores were collected from the NIOZ cores and incubated with overlying bottom water to assess fluxes of TOxN and nitrite, ammonia, silicate and phosphate in the absence of direct flow forcing (herein termed ‘diffusive’) using two similar sampling methods (Trimmer et al. 2005; Mayor et al. 2012; Main et al. 2015). Sub-samples taken from the overlying water provide a time-series of nutrient exchange, and data presented here are combined from between 5 and 11 cores spanning all three SSB cruises that took place in 2015 (Table 5, Online Resource 1, Online Resource 6). Fluxes are stated with reference to the sediments (i.e. a negative result indicates removal from the water column overlying the sediment). Where there is no measurable change in nutrient concentrations, the flux is quoted as zero. Data are not corrected for water column controls (overlying bottom water in the absence of sediments).

On average, the fluxes of all macronutrients are positive, indicating a general release of macronutrients from the sediments into the water column. However, both negative and positive nutrient fluxes are measured at all sites, except for silicate fluxes at site A, which were consistently positive (0.206–3.741 mmol m^−2^ d^−1^). The range of fluxes measured at each site for all nutrients was such that there was no significant difference when considered spatially between sites. Both nitrite and TOxN fluxes are lowest on average at site A and increased through sites I and H, with the highest average fluxes at site G. The greatest range in nitrite and TOxN fluxes are at site H (−0.035 to 0.132 and −0.586 to 0.649 mmol m^−2^ d^−1^ respectively). The fluxes of ammonium are highly variable at all four sites, and site I is the only one to be negative overall with an average flux −0.003 mmol m^−2^ d^−1^. Sites G and H have the highest fluxes of ammonium (>0.04 mmol m^−2^ d^−1^) with the greatest range at site H. Silicate fluxes are on average highest at site A (1.212 mmol m^−2^ d^−1^) almost double that of the other sites. Site H and I silicate fluxes are very similar with the lowest fluxes at site G (0.531 mmol m^−2^ d^−1^). Phosphate fluxes are highest at Site A, which has a negative flux (into the sediment) on average (−0.018 mmol m^−2^ d^−1^) and has the smallest range of fluxes compared to the other three sites.

Diffusive iron (Fe) fluxes are positive at all sites ranging from 0.01 to 54.4 × 10^−3^ mmol m^−2^ d^−1^. Averaged across the year, diffusive Fe fluxes are highest at site A (14.4 ± 19.7 × 10^−3^ mmol m^−2^ d^−1^), and 3-times lower at the site with the coarsest sediments, site H (2.70 ± 5.54 × 10^−3^ mmol m^−2^ d^−1^). However, the range in Fe flux calculations is also greatest at site A, and equal to the range across all sites, while the range is smallest at site H. It is important to note that our assessment of diffusive Fe flux requires a simplification of benthic exchange processes. For example, the roles of advection and bioturbation/bioirrigation at these sites are not accounted for directly in the presented results, and yet they can serve to enhance the transport of Fe (e.g. Reynolds et al. in prep).

#### Variability in biological abundance, biomass and diversity

##### Large mobile epifauna

Note that some shallow burrowing infauna were also collected, but for clarity all fauna collected in the trawls will be termed as epifauna.

At all sites, epifaunal organisms are rather sparsely distributed (Table 6). Average abundance was highest at site G, although differences between sites are not statistically significant. Average blotted wet weight biomass values are lowest at sites I and H, slightly higher at the site G and highest of all at the site A, with significant pair-wise differences between all sites (p < 0.01) except between A and H or G. Diversity is highest at H, with site G being just a little less diverse. Sites A and I has the lowest epifaunal diversity.

Autosub3 seabed photographs were also analysed to estimate faunal density and biomass during DY034. At the time of survey, near-bottom water column turbidity at Site A prevented the acquisition of useful seabed photographs. All megabenthos and demersal fish were counted, measured and identified to the lowest taxonomic level possible (Table 6; Example images can be found in Online Resource 7). For comparability with trawl-caught megabenthos biomass data, our estimates are scaled to a sampling unit equivalent to trawl catch data (500 m^2^). Three phyla dominated the three sites: (1) Cnidaria are the most dominant at Site I and H and the third dominant at Site G; (2) Arthropoda is the second dominant at all sites; and (3) Echinodermata is the dominant at Site G and the third dominant at Site H and I.

#### Mega-infauna (>1 cm)

All sites contain very few large infaunal species with no single sample containing more than a couple of individuals. It is concluded that, due to their low densities, large (>1 cm) infaunal organisms are not a substantial part of the benthic fauna in the study area and that adequate sampling of the benthic fauna is provided by the Jennings trawl (large epifauna) and the 0.08 m^2^ NIOZ boxcorer (macrofauna).

#### Macro-infauna (>1 mm)

Macrofaunal abundance is highest at sites I and H. Site A has slightly lower average abundance, significantly lower than H and G (p < 0.05) whilst site (G) has less than 50% of the abundance of the other three sites (p < 0.0001).

In direct contrast to abundance, wet weight biomass (g m^−2^) is considerably (2–3×) higher at site A than at the other three sites. This indicates that the average body size of macrofauna is larger at site A than at the other three sites.

The average number of species per 0.08 m^2^ core (a measure of α-diversity) is highest in the intermediate sites H and I, with significantly lower diversity seen at sites A (p < 0.001) and G (p < 0.0001). However, the cores taken at site G are much more variable in terms of species composition and this higher variability in species between replicate samples (β-diversity) meant that the total number of species identified at site G is the same as site I and only a little less than site H. Site A displays relatively low diversity compared to the other sites.

Macrofauna abundance and biomass data were combined with published trait information describing modes of sediment reworking and mobility (Queirós et al. 2013) to calculate the average community bioturbation potential (BPc) for each of the sites following Solan et al. (2004). Whilst BPc is not a direct measure of the process of bioturbation it does provide a theoretical estimate of the potential of a community to biologically mix the sediment. All of the 4 sites display notably low levels of BPc (mean ± standard deviation) with the highest values of bioturbation predicted for the muddy site A (36.70 ± 22.53), followed by site H (30.31 ± 20.33) and site I (25.01 ± 17.70). The lowest levels of predicted bioturbation are for site G (19.11 ± 13.14). However, the ranges are large.

**Fig. 14:**
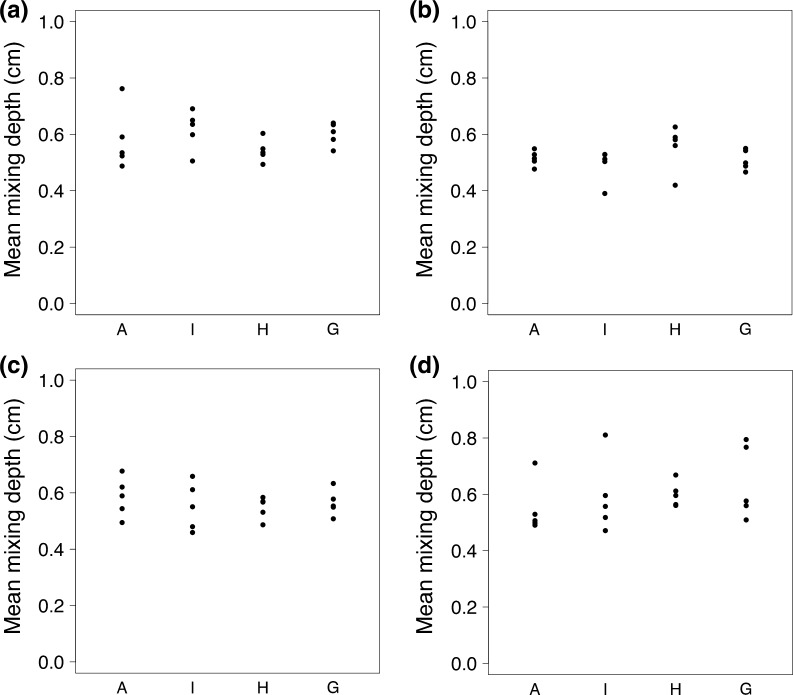
Mean mixing depths across the process sites, associated with macrofaunal infaunal bioturbation: **a** March 2014; **b** March 2015; **c** May 2015; **d** August 2015

Macrofaunal bioturbation activity was measured through quantification of redistribution of fluorescent particle tracers and absolute changes in concentrations of the inert tracer sodium bromide respectively (following Hale et al. this issue). Activity levels are very low (Fig. 14) across the Celtic Sea shelf compared to other UK shelf areas (Dauwe et al. 1998; Teal et al. 2008), and similar across all sediment types observed. The median (^f-SPI^L_med_, typical short-term depth of mixing), maximum (^f-SPI^L_max_, maximum extent of mixing over the long-term) and mean (^f-SPI^L_mean_, time dependent indication of mixing) mixed depths of particle redistribution are presented in Table 6. In addition, the maximum vertical deviation of the sediment–water interface (upper–lower limit = surface boundary roughness, SBR) provides an indication of surficial activity. Bioturbation is heavily influenced by the presence of mobile active species, such as *Nephrops norvegicus* and *Goneplax rhomboides*. Bioturbation activity is observed to peak in August with sediment surface mixing occurring to a depth of approximately 8 mm.

#### Meiofaunal nematodes

Note that only data from the first two cruises (DY008 and DY021) are presented here.

Meiofauna at site A is most abundant with average densities over 800 × 10^3^ Ind m^−2^ and maximum values of >1200 × 10^3^ Ind m^−2^. Sites I, G and H are very similar in terms of meiofauna abundance, with average values lying between 550 and 600 × 10^3^ ind m^−2^, however the differences are significant (p < 0.05). Muddy sediments are known to harbour greater densities of nematodes (Steyaert et al. 1999), the dominant meiofauna phylum with 85.6% (65.3–97.6%) of total abundance, so the high densities at site A are likely a reflection of sediment composition and related interstitial space (i.e. greater porosity in muddy sediments at site A, Table 5) available to meiofaunal organisms. These values lie within the range of densities commonly found in marine subtidal areas (Heip et al. 1985).

In terms of biomass (based on nematodes) site A and I are very similar (1.13 ± 0.35 and 1.14 ± 0.48 g wet weight m^−2^, respectively; p = 0.97), and G and H are similar (0.68 ± 0.17 and 0.73 ± 0.39 g wet weight m^−2^, respectively; p = 0.701). As with abundance values, biomass values lie within the ranges observed for European subtidal areas (Heip et al. 1985) with distinct differences between muddy and sandy sediments. All pairwise comparisons between sites A, I and G, H results in significant biomass differences (p < 0.05).

On the phyla level, multivariate meiofauna community structure data is significantly different between sites and seasons (p ≤ 0.01), and, like abundance and biomass, considerable similarity was found for site pairs A and I (p = 0.635), and G and H (p = 0.054), whilst all other pairwise comparisons show significant differences (p ≤ 0.05).

#### Microbes

**Fig. 15:**
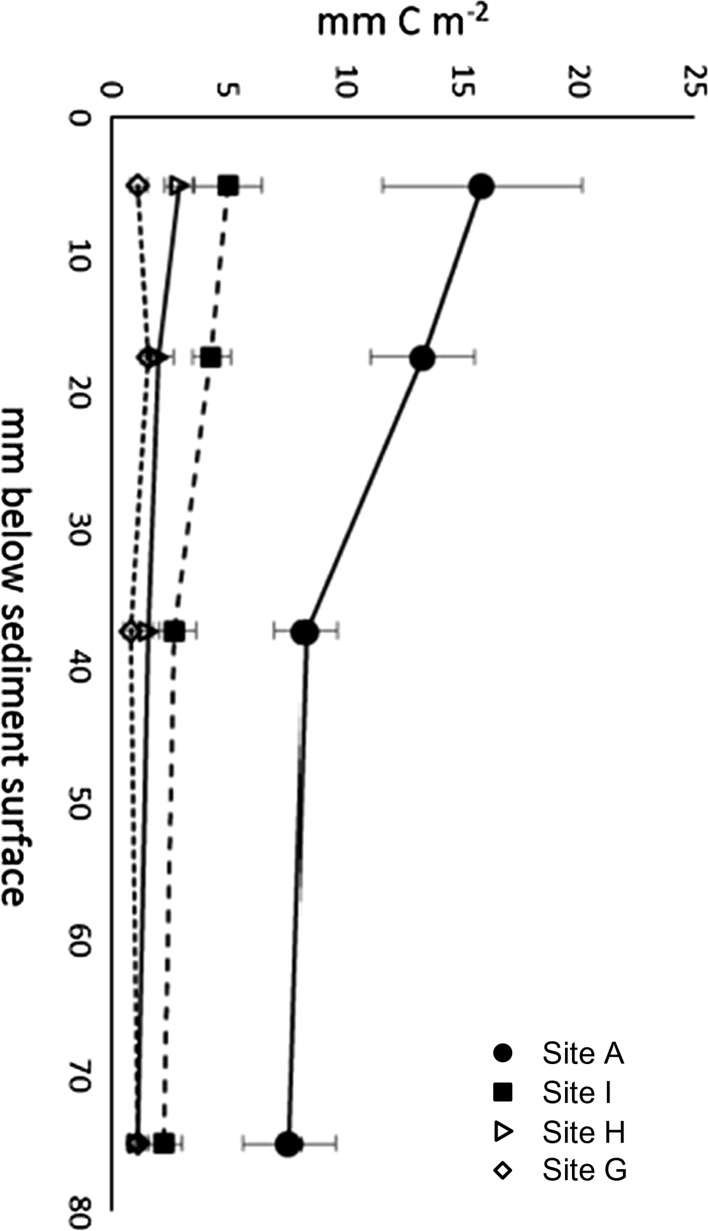
Microbial biomass (mm C m^−2^), estimated from direct counts of microbes. Station A *filled circles*, station I *filled squares*, station H *open triangle* and station G *open diamond*. *Error bars* are standard deviation

Porosity (Table 4) is a major determinant of microbial biomass, with the highest measurements at site A and the lowest measurements at site G (Fig. 15). Biomass decreases with sediment depth for all except site G.

Bacterial 16S rRNA genes dominate the total microbial assemblages within coastal sediments, with reports of only 2% of 16S rRNA genes affiliated with archaea (DeLong 1992). Our data suggest a higher abundance of archaea in shelf sediments, in all sediment types examined, with little evidence of differences in the ratio of archaeal:bacterial 16S rRNA genes with depth. At site A, 29.7% (±16.5) of 16S rRNA genes are archaeal, and at site I this figure is 35.8% (±15.9), 38.3% (±20.9) at site H and 22.2% (±14.2) at site G; the differences between sites are significant (p < 0.05).

### Step 3b summary

Habitat variations across the four sites echo the differences in sediment variation seen within the constrained target area, and confirmed that the process study sites represent significantly different habitats. These differences were also reflected in the bulk biogeochemical properties of the bed, although seasonal variability in pore water concentrations and nutrient fluxes are sufficient to mask spatial variability between the sites.

## Discussion

We have described the way the four process study sites, which encompass the range of sediment and habitat variation seen in UK shelf seas, were identified within a constrained target area of the Celtic Sea, for investigation within the benthic component of the SSB programme. The sites differ significantly in terms of sediment, habitat type and bed structure, whereas differences in confounding physicochemical variables are minimal and seasonal changes (e.g. the phenology and magnitude of the spring bloom) occurred concurrently across the sites. This provides discrete, exemplar process study sites across the appropriate range of bed types to represent the wider region, for targeted field campaigns as part of the SSB programme.

Logistical limitations to in situ observations, sampling and experimentation are unavoidable, and decisions must often be made early in the project planning stages regarding site selection. In shelf sea environments, which are both spatially and temporally variable at a range of scales, this site selection process becomes particularly important; especially where results are intended to be up-scaled and used to represent or model systems at shelf or regional scales.

In these cases, as in the SSB Programme, the key to addressing such issues is to consider these scaling necessities from the outset, and to assess regional scales and variability during the site selection process (e.g. Painting et al. 2013; Savchuk 2002). Thorough evaluation of the previously available datasets is paramount to ensure that what are often limited resources can be put to best use to address the scientific questions being asked.

It is apparent that neither observations nor models in isolation are sufficient for a regional assessment of benthic biogeochemical cycling; observationalists and modellers working together can improve process understanding and scaling processes (e.g. Steiner et al. 2016; Queirós et al. 2015). Some of the key points to consider during the site selection process are: the representativeness of any data collected to the desired model outputs (Steiner et al. 2016); the number of observations needed to address key uncertainties that affect existing parameterisations; the identification of processes not currently considered (Steiner et al. 2016); and the benefits of interdisciplinary/holistic approaches to parameterisation (Queirós et al. 2015).

The methodology presented here is therefore to first assess shelf-scale variability in order to step-down in scale to the local and then site scales consistent with the scientific requirements and technical restrictions of the project. This allows a clear pathway forward for the subsequent upscaling required for shelf scale assessments of biogeochemical cycling, in contrast to site selection based on isolated bed or local variables alone.

### Site selection considerations

#### Spatial heterogeneity

Three scales of heterogeneity were assessed within the site selection process: shelf-; local- and site-scale. These were assessed using a combination of existing data and models (shelf scale—Stephens 2015; Stephens and Diesing 2015); observation (local scale—spatial survey; landers and buoys; Autosub); and replication (site scale). Limited resources typically preclude the assessment of shelf-scale heterogeneity directly through observation and therefore necessitate the use of existing data, e.g. the British Geological Service (BGS) surface sediment database (DigSBS250). The use of extant data has inherent limitations, including: temporal differences in sample collection; variable resolution; and methodological differences in data collection or analysis. Nevertheless, these data present a reasonable representation of the variability of the shelf sediments, if not an exact map of their current extent and location. In combination with scaling approaches such as Stephens and Diesing (2015), this provides sufficient overview for the selection of a targeted region. At the local scale, spatial surveys, such as the one carried out here, can be used to ground truth existing sediment maps, giving additional confidence in the data that will subsequently be used during the up-scaling process. Such surveys can generate large numbers of samples, restricting the number of stations that can be visited and limiting replication, so a balance between resolution and resources is necessary. At the site-scale, variability can be at the scale of mm to dm and the range of measurements and experimental techniques being made often target different scales (for example O_2_ profiling at the μm to mm scale versus in situ flume deployments at m^2^ scales). To address this, sufficient replication is required to determine the variability within the data, in order to interpret whether any temporal/seasonal changes observed fall within the natural spatial variability of the sites (Mouret et al. 2016).

In terms of the SSB work considered here, this process allowed a relatively simple justification to be made for the selection of the process sites. The targeted area was determined based on a balance of maximum sediment heterogeneity and minimum confounding variable complexity. The assessment of the spatial variation within the targeted area (1) justified the use of the surface sediment coverage model (presented in Figs. 1, 4, 8), (2) allowed an assessment of the representativeness of the area in comparison with the shelf as a whole, and (3) provided baseline values of this variability with which to make the final site selection.

#### Assessments of confounding variables

Throughout the selection process, it was essential to maintain a clear focus on the scientific objectives of the programme, set out in the overarching aims of the SSB programme. However, the shelf is a complicated system, and local environmental conditions such as bottom water temperature, oxygen and nutrient concentrations and pelagic primary production inputs are all known to affect biogeochemical cycling within shelf sediments (e.g. Soetaert et al. 1996, 2000; Dollar et al. 1991; Wijsman et al. 1999; Van Cappellen et al. 2002; Fulweiler et al. 2010; Dale et al. 2011). Because the focus of the SSB work is on bed type, these local conditions are considered confounding variables, which can be a particular problem when smaller-scale variables are extrapolated (Morrisey et al. 1992). The focus was therefore to minimise any differences in these variables between the sites, so as to simplify analysis, and avoid the risk of masking the signals of interest. In our case, the hydrodynamic variables, timing and onset of stratification, and the phenology and magnitude of the spring bloom (Chlorophyll and CO_2_-drawdown) were similar across sites, thereby minimising the impact of these confounding variables.

#### Minimum site and visit numbers

Deciding upon the number of sites that will be visited and the frequency of those visits requires careful consideration of, amongst other things, necessary replicability, the importance of spatial versus seasonal variability, and the scope of observations; as well as restrictions on ship time, manpower and available funds. The resulting selection must reduce the number of sites to what is logistically achievable whilst maintaining the delivery of the required scientific outcomes of the project. In the case of the SSB programme, the key importance of the spring bloom on the biogeochemical processes (Zhang et al. 2015) dictated the temporal visitation requirements (minimum of 3 visits: pre-, during- and post-bloom); while the variations in sediment type were the key factor considered in terms of spatial requirements (see “Step 3a” and “Assessments of confounding variables”). As a minimum, the end-member conditions for a given parameter within the region must be investigated, ideally with information at intermediate sites to ‘fill in the gaps’. Given the range of sediments present in the Celtic Sea area, the chosen end members were sandy mud (>50% fines) and sand (<15% fines). Two additional intermediary sites representing fines percentages of ~20 and 30% were considered sufficient to provide an overview of the region, and represent a gradient between the end-members. This resulted in the minimum requirement of four sites, and twelve site visits over the lifetime of the programme. To illustrate the scale of this programme, it should be noted that each ‘site visit’ resulted in the collection of approximately 60 NIOZ cores; 5 SMBA cores; 3 Megacores, trawls, CTD casts, water column samples, buoy and lander maintenance and deployment, experimental deployments and autonomous surveys.

#### Considerations for data interpretation

It is important to consider the following when interpreting the data collected from these sites and shelf seas in general.

##### Sediment versus habitat type

While the terms are often used interchangeably, they are commonly closely related (LaFrance et al. 2014; Heip et al. 1985), and the faunal analysis performed herein shows that sediment size is generally a good predictor of macrobenthic community structure (McCelland et al. 2016). It should be noted that considerable overlap occurs in species occurrence between closely related sediment types. Hence, habitat and sediment type, while closely correlated, are referred to separately here. While several species showed a strong site preference, there was considerable overlap of several species abundance at several of the sites. A full discussion of species abundance and site preference can be found in Online Resource 8.

##### Seasonality

While a full discussion of seasonal signals in the data is beyond the scope of the present manuscript, which aims to detail the site selection procedure and present overall ranges for the measured parameters, it is clear that an analysis of temporal variability associated with the bloom conditions is key to realising realistic biogeochemical budgets on the shelf. In particular it was noted that temporal variability could lead to large ranges in some biogeochemical parameters, which can mask spatial differences. More details of these seasonal trends can therefore be found within the other contributions to this special issue, or through direct analysis of the data (see Online Resource 1 for details of how to obtain this data).

##### Anthropogenic influences

Marine observations and experiments often aim to investigate conditions relative to a defined baseline condition, to quantify change (Franco et al. 2015). The UK shelf seas are under the influence of significant present and historical anthropogenic pressures, which prevent a no-influence baseline being established, and it is often difficult to predict how these pressures may have or will change over time. Best practice is therefore to establish the historical influences that occurred before the study, monitor those that occur during it, and interpret the results with these in mind. The anthropogenic influences are varied, and we will not consider all of them here, however, the effect of trawling has the largest spatial impact directly on the seabed, and we briefly discuss this below.

##### Trawling pressures

**Fig. 16:**
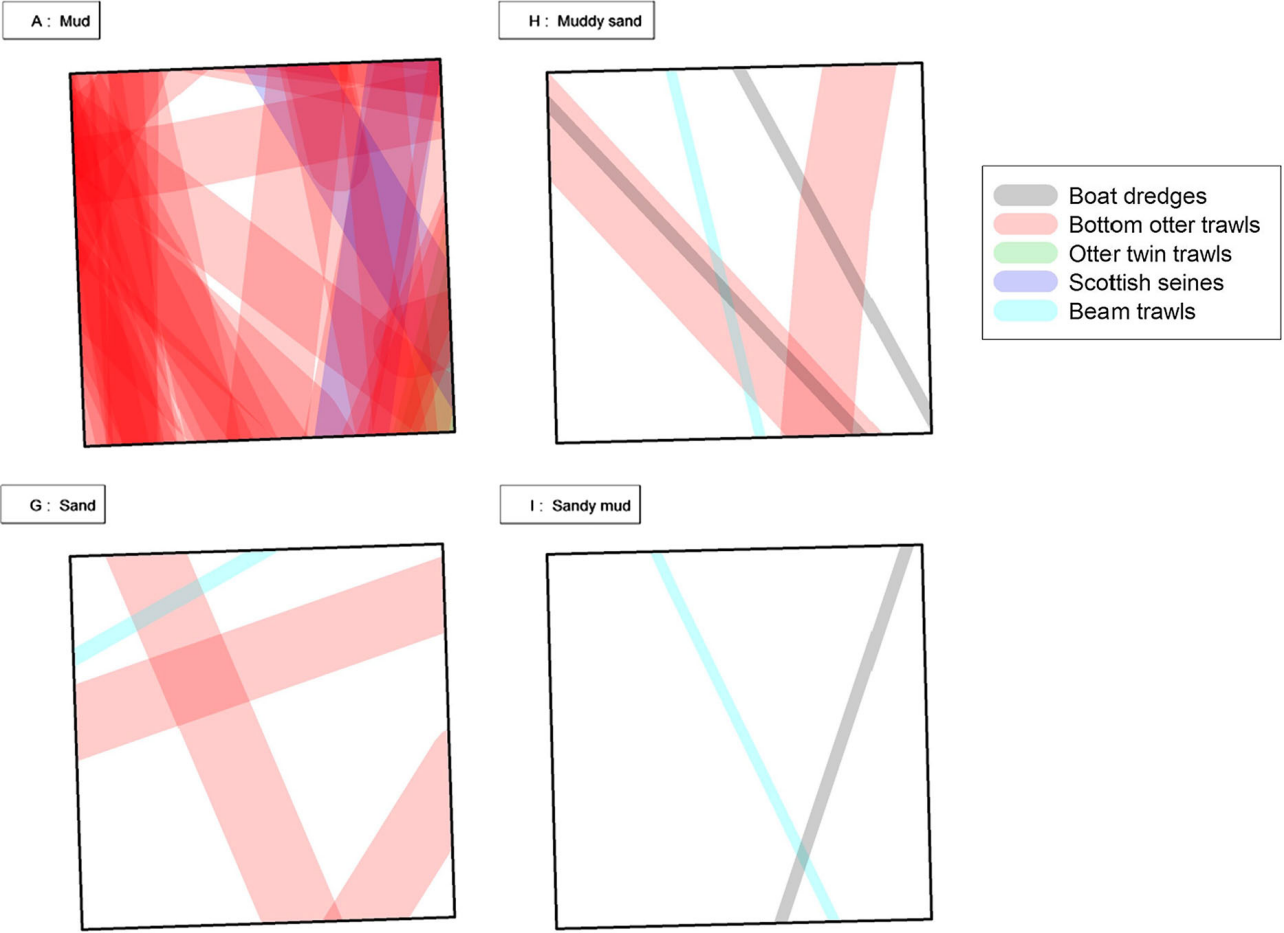
Trawl tracks across the four process study sites (500 × 500 km, represented by the *black squares*) between March 2013 and August 2015, indicating frequency and width of trawl tracks

Commercial fishing is extensive in our chosen sampling region, and many fishing techniques have a considerable impact on the bed. Trawling is intense and frequent in box A (Fig. 16), with only a minor fraction not trawled in the period from March 2013 to August 2015. On average, the entire box is trawled 4.23 times over this period. The main gear used was otter trawls. The doors of otter trawls (and clumps for otter twin trawls) can penetrate the sediment to depths up to 35 cm (Eigaard et al. 2015), but the sweeps and ground rope will not penetrate more than a few cm. Trawling is less intense in boxes G and H with only half of the box being trawled, and virtually absent in box I, which is mirrored by the sidescan survey data presented (Fig. 10; Online Resource 5).

This is only part of the story, however. In order to estimate whether benthic trawling had impacted noticeably on the structure of macrofaunal communities we calculated the average AZTI Marine Biotic Index (AMBI) for each of the four process sites. This index is derived from the relative distribution of individuals across five ecological groups spanning a range of sensitivities to disturbance (Borja et al. 2000). The index is designed to calculate values that fall along a continuum from 0 (a community completely dominated by sensitive species and therefore undisturbed) and 7 (a completely azoic sediment). Our data indicated that despite the high frequency of trawling identified at some of the sites, AMBI scores were generally low, with the highest average score of 2.25 (±0.54) being recorded at site A as expected. For the other sites the AMBI scores were all lower, and within similar ranges (site I, 1.01 ± 0.40; site H, 0.74 ± 0.29; site G, 1.12 ± 0.31). This suggests that benthic trawling may have only a minor impact on the structure of the macrofauna at 3 of our sites, and only at site A is there evidence that the communities are even slightly disturbed. Consequently, the relatively low levels of macrofaunal abundance, biomass, biodiversity and bioturbatory function seen at all our sites must be driven by some other factor or factors. For the meiofauna, there is no indication for trawling disturbance at the phylum level given the high abundance at site A and the community similarity between A and I. We expect, however, that the physical disturbance will be evident in nematode genera/species data since previous studies have documented that physical stress, such as trawling, impacts nematode diversity, function and community structure (Schratzberger et al. 2009; Schratzberger and Jennings 2002).

Trawling in a region can have an additional indirect impact on long-term studies such as this one: both the NB and ECD landers were lost during June 2014, likely through trawling activities. When they were relocated in October 2014, a new site was chosen (CD2L) which gained protection from a known long term monitoring position of which fishermen were aware.

## Future pressures

An additional consideration when interpreting the data collected in a programme such as this is that data collection focuses on a limited window of time—in this case a little over a year. Spatial patterns are likely to change over time, and the interactive effects of spatial and temporal changes are likely to mean that each site evolves along a different trajectory (Morrisey et al. 1992). The SSB programme design is sufficient to capture seasonal cycles, but not climatic ones. We must consider that longer scale temporal changes would have an effect on any future scenario modelling or prediction, and that we are not able to capture that in the field. Our approach is to determine where the sampled ‘year’ fits against the typical conditions experienced on the shelf, and use experimental and laboratory work to investigate this.

## Conclusions

The Shelf Seas Biogeochemistry programme set out to assess the importance of the key variables of sediment type and seasonality on carbon and nutrient cycling in UK shelf seas. As part of this programme, exemplar sites for mechanistic and deterministic measurements of benthic biogeochemical processes were identified on the basis of their potential to aid future up-scaling activities to the shelf-scale. Our observations and activities will increase our broad-scale understanding of benthic biogeochemical processes and improve our predictive shelf-scale modelling capabilities.

The choice of our study sites is based on a three-step selection process in which the regional context of the UK continental shelf is the main focus. Initially, a constrained target area within the Celtic Sea was chosen to be representative of the sedimentary heterogeneity encountered across the wider UK shelf. This also provides a focal region for long-term observations, cruise operations and in situ experimentation. Secondly, a detailed assessment of the spatial and temporal heterogeneity within this target area was made. Lastly, four process study sites were chosen within this region which captured the necessary range of benthic variability needed to address the scientific focus of the benthic component of the SSB programme.

Assessment of this procedure has led to the following recommendations:

Step One: The initial choice of a targeted region of operations must allow a careful balance between resources and scientific requirements. Sufficient variability in the key scientific variables should be ensured, as well as a reduction in the potential effects of any confounding variables, and minimisation of the overall size of the operational area for logistical purposes.

Step Two: A full assessment of the variability within this target area allows:

- Confirmation of sufficient spatial heterogeneity;
- Assessments of the targeted region within the context of the wider continental shelf (i.e. is the region representative?);
- Determination of whether existing, larger scale models and predictions of shelf-scale heterogeneity (used in step 1) are accurate; essential for subsequent up-scaling.

Step Three: The final choice of process study sites requires them to:

- Fully encompass the range of spatial heterogeneity occurring across the target area;
- Be sufficiently different in terms of the key scientific variables;
- Be sufficiently similar in terms of confounding variables;
- Be small enough to minimise within-site heterogeneity, which can then be addressed through sufficient replication;
- Have sufficient replication across scales to have sufficient statistical power to find hypothesised differences among metrics.
- Be large enough to reduce over-sampling during repeat, seasonal visits.

In relation to the SSB programme, following the above procedure led to the selection of four exemplar process study sites that span the full range of variability exhibited on the UK shelf. These sites are significantly different in terms of their sediment and habitat type, yet are highly similar in terms of confounding variables e.g. hydrodynamic forcing, water depth, temperature, and salinity. We contend that the proposed site selection procedure ensures a very strong likelihood of site-specific work being useful for up-scaling activities and thus increasing our understanding of benthic biogeochemistry at the UK-shelf scale.

## Electronic supplementary material

Below is the link to the electronic supplementary material.

Online Resource 1 Detailed methodologies and data availability (DOCX 175 kb)

Online Resource 2 Long Term Observation deployment positions and operation timescales (DOCX 78 kb)

Online Resource 3 Spatial survey sediment characteristics, organised by  % Fines < 63 μm (DOCX 174 kb)

Online Resource 4 Bathymetric maps generated from Autosub3 (a) site A, (b) site G, (d) site I and Autosub6000 (d) site H multibeam data (smoothed at 50 m horizontal scale). Water depth ranged from 101-106 m at Site A, with the study box having a general depth of 103 m; 96-101 m at Site G, study box general depth of 98 m; 106-107 m at site I, study box general depth of 107 m; 103-109 m at Site H, study box general depth of 105 m (TIFF 4187 kb)

Online Resource 5 Sidescan of the 4 study sites: (a) Site A, (b) Site G, (c) Site H and (d) Site I. All scale bars represent 100 m. The parallel white lines are the nadir and represent lines with no data. Note that at Site G, the white vertical band represents an area where no data were collected. The presence of repeating backscatter ‘stripes’ at Site G is clear and appear to be matched by bathymetric variations suggestive of sedimentary bedforms. Presumed “trawl marks” (seabed scars resulting from commercial bottom trawling operations) are particularly notable at Site I, but also present at sites A, G and H (TIFF 5871 kb)

Online Resource 6 Mean Nutrient Fluxes (mmol.m^−2^.d^−1^) averaged over all seasons: Boxes represent mean and SE, with max and min whiskers (DOCX 783 kb)

Online Resource 7 Example of density dominant fauna across sites G, H and I. Taxa were determined to the lowest taxonomic level whenever possible (otherwise a morphotype was assigned). Arthropoda (a) *Nephrops norvegicus* and (d) *Goneplax rhomboides*; Echinodermata Asteroidea (b) *Astropecten irregularis*, (f) *Luidia sarsii;* and, *Cnidaria* (c) *Cnidaria spp*. type 01, (f) *Bolocera spp*. type 01 (TIFF 4930 kb)

Online Resource 8 Site specific species abundance (DOCX 125 kb)
