# Supplementary material for: An approach for the identification of exemplar sites for scaling up targeted field observations of benthic biogeochemistry in heterogeneous environments
Source: Biogeochemistry. 2017 Aug 1;135(1):1–34. doi: 10.1007/s10533-017-0366-1 (PMC6961521; doi:10.1007/s10533-017-0366-1)
Supplement: Supplementary file 1 — Detailed methodologies and data availability (DOCX 175 kb) [file 10533_2017_366_MOESM1_ESM.docx]

Online Resource 1: Detailed Methodologies and data availability.

**Water Column Observations and Sampling**

| **Benthic Landers.** |  |
| --- | --- |
| *Continuous Monitoring*: | A series of benthic landers were designed by the Centre for Environment, Fisheries and Aquaculture Science (Cefas) for continuous monitoring of near-bed water column parameters. They were equipped with an ESM2 logger (Cefas, UK) recording in bursts of five minutes every 30 at a sampling frequency of 1 Hz, from the following sensors: *Aanderaa 3919B:* Conductivity (range 0-7.5 S/m; Resolution 0.0002 S/m; Accuracy ± 0.0018 S/m) and Temperature (-5 to +40^o^C; Resolution 0.01^o^C; Accuracy ±0.1^o^C); *Druck PDR 1828*: Pressure (20bar operating range; Accuracy ±0.1%); *Seapoint STM*: turbidity (Sensitivity range 25-4000 FTU depending on gain); *Aanderaa Optode 3835*: Oxygen Saturation (Range 0-500μM; Resolution <1 μM; Accuracy <8 μM or 5%); *Seapoint SCF*: chlorophyll fluorescence (Sensitivity range 5 – 150 μg/l).  Additionally, an upward facing *RDI 600 kHz workhorse ADCP* (Range ±10m/s; Resolution 0.1cm/s; Accuracy ±0.3% ±0.2cm/s) recorded in burst mode for five minutes every hour at a sampling frequency of 1 Hz. |
| *Intra-tidal Monitoring:* | The National Oceanography Centre (NOC) Liverpool designed miniSTABLE lander allowed shorter-term, higher frequency intra-tidal monitoring of near-bed properties. This was equipped with the following sensors: Top mounted *RDI 1200 kHz ADCP*: Velocity, Backscatter (Range ±10m/s; Resolution 0.1cm/s; Accuracy ±0.2% ±0.1cm/s); Bottom mounted Unisense *oxygen eddy correlation system* (Nortek Vector ADV (Range 0.01 – 7m/s; Accuracy ±0.5% ±1mm/s), Unisense oxygen microsensor (Range 0-1 atm pO_2_; Detection limit 0.3 μM), and Aanderaa oxygen optode (Range 0-500μM; Resolution <1 μM; Accuracy <8 μM or 5%)); *Aquascat Acoustic Backscatter Sensors* (1,2,3,4 MHz: Range 0.01 -20g/l); *3D ripple profiler* (1.1 MHz dual axis pencil beam scanning sonar (Thorne and Hanes, 2002; Marine Electronics, 2009)); *Nortek Aqua-Dopp HR* (2 Mhz; Range ±10m/s; Accuracy 1%±0.5cm/s); *LISST-Holo* (Range 25-2500 μm); *LISST 100X*, (Range 2.5 -500 μm and 1 – 900mg/l; Resolution < 1mg/l); *McLane RAS* water sampling system (48 x 500ml samples); *FSI CTD* (Temperature accuracy ±0.0002 S/m; Conductivity accuracy ±0.005^o^C; Pressure accuracy ±0.08%); and *Satlantic SUNA nitrate sensor* (Range 4000μm; Accuracy ±2 μM; Limit of detection 2μM). |
|  |  |
| **Buoys** | Cefas designed *SmartBuoys* provided a long term high-frequency time series (at 1 m below sea surface) of salinity, temperature, turbidity, oxygen saturation, chlorophyll fluorescence (sensors and sampling regime as for Continuous Monitoring Landers). A water sampler (Cefas Technology Ltd (CTL), UK) collected unfiltered samples into parenteral nutrition bags pre-spiked with mercuric chloride for subsequent nutrient analysis, either immediately, or within 30 days of collection. A quantum irradiance sensor (LiCor LI-192: calibration ±5%; Sensitivity 4μA; Response time 10μs) mounted just above the sea surface provided long term measurements of the photosynthetically active light climate for the area. |
|  |  |
| **Underway data** | Underway pCO_2_ data were collected using the PML-Dartcom Live pCO_2_ system during the cruise programme, sampling atmospheric pCO_2_ and headspace equilibrated seawater pCO_2_ every 20 minutes and calibrated against NOAA-traceable CO_2_ standards with an accuracy of ±4 μatm (Ribas-Ribas *et al.*, 2014). Underway chlorophyll *a* was determined fluorometrically and calibrated against known chlorophyll *a* standards (Welshmeyer, N. A., 1994). |
|  |  |
| **CTD** | Water column profiles of temperature, salinity, depth, chlorophyll fluorescence and turbidity were collected, along with water samples for sensor calibration and nutrient analysis using both standard and titanium (ultra-clean) Sea-Bird CTD systems. Both the standard stainless and the titanium systems used: Sea-Bird 9plus underwater unit, Sea-Bird 3P temperature sensor, Sea-Bird 4C conductivity sensor, Digiquartz temperature compensated pressure sensor, Sea-Bird 5T submersible pump, s/n 05T-5247, (primary) Sea-Bird 5T submersible pump, Sea-Bird 32 Carousel 24 position pylon, Sea-Bird 11plus deck unit, Sea-Bird 43 dissolved oxygen sensor, WETLabs light scattering sensor, Benthos PSA-916T altimeter, Chelsea Alphatracka MKII transmissometer, Chelsea Aquatracka MKIII fluorometer, Biospherical QCP Cosine PAR irradiance sensor and a Biospherical QCP Cosine PAR irradiance sensor. |
|  |  |
| **Fishing Activity** | Accurate assessments of the amount of different fishing activities and their intensities, and potential effects is difficult. Nevertheless, estimates can be made using AIS (Automatic Identification System), which was introduced by the International Maritime Organisation (IMO) in the 1990s to improve maritime safety and avoid ship collisions (Natale et al*.*, 2015; McCauley et al., 2016). All vessel positions in an area of 2nm around the process study sites were obtained from a satellite derived AIS dataset (S-AIS) for the period between March 2013 and August 2015. This dataset contains vessel positions at intervals of ~15 minutes, and vessels fishing at a speed of less than 8 knots should therefore leave at least two records within each area. Fishing vessels, their main gear type, engine power and overall length were obtained by matching their MMSI from the AIS data to the EU fleet register (<http://ec.europa.eu/fisheries/fleet/index.cfm>). Only actively towed bottom fishing gears were considered in this analysis. The speed of vessels was calculated from the distance and time between subsequent records, and fishing was assumed to occur between 1.5 and 5 knots. Trawl tracks were reconstructed by connecting AIS records from individual vessels where the record sampling interval was <20 minutes. The width of the trawl gear was calculated from the engine power or vessel length using relationships given in Eigaard et al. (2015). Trawling is also evident from Autosub sidescan imaging (Figure 10; Online Resource 4). |

**Benthic Sampling**

| **Autonomous Underwater Vehicle Survey** | The *Autonomous Underwater Vehicles (AUVs)* Autosub3 and Autosub6000 (e.g. Morris et al, 2014) were used to survey the process study sites. At each site the AUV surveyed between 3 and 6 transect lines 5 km in length, with a line spacing of ~150 m, at a nominal speed of 1.4 ms^-1^. Data collected included: i) swath bathymetry (50 m altitude) using a Kongsberg EM2000 multibeam system (200kHz frequency, 111 roll stabilised beams per ping; 1.5^o^ x 1.5^o^ beamwidth with 150^o^ angular coverage); (ii) sidescan sonar (15 m altitude) using an EdgeTech 2200-FS (75 – 600 kHz); and (iii) photography (~3.2 m altitude) using a Point Grey Research, Grasshopper2, mounted vertically downward. |
| --- | --- |
|  |  |
| **Coring** | Principal sediment sampling was carried out using a *NIOZ (Haja) Boxcorer (K16)* with 320 mm diameter cylindrical core barrels, providing a 0.08 m^2^ core sample with overlying water. In many cases these were then sub-sampled to provide specific sized cores or sediment samples for subsequent experimentation and analysis (described in more detail below where necessary). Larger sediment samples for faunal analysis were collected using an USNEL-type 500 mm square (0.25 m^2^) *Scottish Marine Biological Association (SMBA) Box Corer*. A Bowers and Conelley *Megacorer* was used to take multiple (up to 12) simultaneous sediment samples in 100 mm diameter pre-drilled polycarbonate core tubes up to 300 mm in length, from an approximately 0.25 m^2^ area of the seabed for iron pore-water analysis (Barnett et al., 1984; Aquilina, 2014; Homoky et al., 2013). |
|  |  |
| **Pore Waters** | Samples of pore water nutrients were collected using a novel in situ device developed at Cefas and described in detail elsewhere (e.g. Duplisea et al., 2001; Trimmer et al., 2000; Trimmer et al., 2005; Sciberras et al, 2016; Weston et al., 2008). Porewater iron (Fe) was extracted at 1 – 2 cm depth intervals using Rhizon filters (0.15 μm), inserted into pre-drilled holes in custom Megacorer tubes (Seeberg-Elverfeldt et al., 2005; Homoky et al., 2013; Klar et al., this issue). |
|  |  |
| **Diffusive Nutrient Fluxes** | Five 10 cm diameter sediment sub-cores were collected from separate NIOZ cores at each site. Overlying water in each core tube was topped up to ~1000 ml with collected site bottom water and the cores placed in a thermostatically controlled water bath set at the bottom water temperature measured on site. The cores were gently aerated and stirred to prevent oxygen depletion. Three core tubes filled with only 1000ml bottom water acted as a bottom water control. After an equilibrium period, sub-samples of the overlying water were removed using a syringe at roughly hourly intervals for the first one to six hours, and then at approximately 12 – 18, 24 and 36 hours after t = 0 to complete the time series. All samples were immediately filtered through a 0.2μm syringe filter and placed in an airtight vial for analysis according to nutrient sample analysis described below. Concentrations were corrected for the volume of overlying water, and the fluxes calculated from the slope of the linear portion of a time series and converted to a flux in μmol.m^-2^d^-1^. |
|  |  |
| **Sediment Profile Imaging** | A *Sediment Profile Imaging (SPI)* camera, manufactured by Ocean Imaging Systems, was used to capture in-situ vertical profile images of the top few centimetres of the seabed, including the sediment-water interface. This is a photographic technique (Rhoads & Cande, 1971; Germano et al, 2011) where a mirrored prism is driven vertically, by its own weight, into the sediment profile and photographs intersecting the sediment-water interface are obtained 15 and 30 seconds after penetration. It used a Nikon D100 digital camera (F10, 1/60th second, ISO400) with a 35 mm lens and self-contained strobe flash unit. |
|  |  |
| **Biological sampling** | |
| *Large mobile Epofauna* | A Cefas 2 m *Jennings beam trawl* was used for the collection of large epifauna; species from 3 replicate 5 minute trawls carried out a ship speeds of 1.5 knots at each of the 4 sites, on each of the three 2015 cruises. The trawl is fully described in (Jennings et al, 1999) and consists of a 60 mm square section beam with chain mat 2 m wide at the mouth, and a 20 mm mesh with a 4 mm knotless mesh liner. Autosub3 seabed photographs were also analysed to estimate faunal density and biomass during DY034. Linear measurements were made to estimate the biovolume of individual specimens and were converted to wet mass assuming unit specific gravity (Morris et al., 2014; Durden et al., 2015). In the case of colonial and encrusting organisms, these were measured as single entities. |
| *Mega-Infauna* | At each site five replicate SMBA boxcores were collected and the sediment sieved over a 1 cm mesh to collect mega-infauna. |
| *Macro-Infauna* | A 0.08m^2^ NIOZ box corer was used to collect 5 replicate cores at each of the 4 sites, on each of the 4 cruises. These cores were sieved over a 1mm mesh and the macrofauna retained were identified, counted and weighed. |
| *Meiofauna* | At all sites and during all four cruises, meiofauna (nematodes >63 µm) was subsampled from the 0.08m^2^ NIOZ box in three 50 ml syringe cores (2.8 cm diameter, approx. 10 cm deep). These were pooled and preserved in 10 % borax-buffered formaldehyde solution. Meiofauna was extracted using Ludox density separation (Somerfield & Warwick, 1996). Ten percent of each sample was investigated under a stereoscopic microscope, major meiofauna taxa (phyla) were identified (Higgins & Thiel, 1988) and nematodes were picked out and mounted on glass slides (Somerfield & Warwick, 1996). For biomass measurements, nematode width and length were measured using a Leica DM3000 compound microscope and DFC450C camera using Leica imaging software, and converted into wet weight biomass using Andrassy’s formula (Andrassy, 1956) adjusted for the specific gravity of marine nematodes (i.e. 1.13 g cm^−3^) and 12.5% C/wet weight ratio (Heip et al., 1985). |
| *Microbes* | At each site, sediment was sub-cored using either 30 mL (for direct microbial counts) or 50 mL (for molecular analyses) syringe cores. Samples for direct microbial counts were sectioned (0 – 10; 10 – 25; 40 – 60; and 80 – 100 mm), immersed in a 2 % glutaraldehyde solution and frozen at –80 ˚C, whereas samples for molecular analyses were immediately frozen intact at -80 ˚C. Microbial abundance was enumerated using microscopy (Manini & Danovaro, 2006), and biomass estimated assuming an average of 14 fg carbon per microbial cell (Kallmeyer et al. 2012). To quantify the ratio of archaeal and bacterial 16S rRNA genes in each sediment sample, DNA was extracted using the MoBio Powersoil Total RNA Isolation Kit with the DNA Elution Accessory Kit (MoBio, Carlsbad, USA) from sectioned sediment (0 – 10; 40 – 60; and 80 – 100 mm). 16S rRNA gene abundances were quantified using the PCR primer pairs and methods published in Tait et al. (2015). |

**Sample Analysis**

| **Nutrients** | Nutrient water column and pore water samples were all analysed on board using a Bran and Luebbe segmented flow colorimetric autoanalyser following Woodward and Rees (2001). Clean sampling protocols were used to avoid contamination, and analysis and sampling were carried out as close as possible to international GO-SHIP protocols (Hydes et al, 2010). Where the sample concentrations were high they were diluted with low nutrient seawater, to bring them within the analytical range of the analyser. Nutrient reference materials (KANSO Japan) were analysed each day to check analyser performance and to guarantee the quality control of the final reported data. The typical uncertainty of the analytical results were between 2-3%, and the limits of detection for Nitrate and Phosphate was 0.02 µmolesl-1, Nitrite 0.01 µmolesl-1, Ammonia 0.05 µmolesl-1, and Silicate did not ever approach the limits of detection. |
| --- | --- |
|  |  |
| **Iron** | Iron concentrations (both Fe(II) and total dissolved Fe) were determined spectrochemically by measuring the absorbance of the Fe(II)-ferrozine complex formed after the addition of ferrozine (and ascorbic acid for the determination of total Fe) to each sample (Stookey, 1970). Concentrations > 1 μM were analysed in a 1 cm quartz cell on a spectrophotometer (ATI Unicam 8625) and concentrations < 1 μM were measured on a 3000 Series Liquid Waveguide Capillary Cell with a 2.5m optical pathway (World Precision Instruments) (Waterbury et al., 1997). Calibration was carried out by analysis of a series of Fe(II) concentration standards, from ammonium iron(II) sulfate hexahydrate (Sigma-Aldrich, purum p.a. grade), made up in a similar sample matrix. On the spectrophotometer, the limit of detection (LOD, three times the standard deviation of the blank) was 0.3 μM Fe(II) and the blank was 0.25 μM Fe(II). The typical relative standard deviation (obtained by measuring replicates) was 2 % for > 10 μM and up to 5 % below 4 μM. For the LWCC, the LOD was 0.7 nM, the blank was 6 ± 4 nM and the typical relative standard deviation was < 5 %. The method is described in detail in Klar et al. (this issue). Diffusive iron (Fe) fluxes were calculated from porewater concentration gradients across the oxic surface layer by combining a 1-dimensional steady state transport equation with the kinetics of Fe(II) oxidation following previous studies (Homoky et al., 2012; 2013) and is described in detail by Klar et al., (this issue)(Table 5). |
|  |  |
| **Sediment Characterisation** | |
| *Particle Size Analysis* | PSA was carried out following the NMBAQC method using a combination of sieve and laser diffraction (Mason 2011). The sediment was wet-split at 1mm, then the >1mm fraction underwent subsequent dry sieving at ½ Phi intervals down to 1mm, and the <1mm fraction was be analysed using laser diffraction with a Beckman Coulter LS13 320. The sieve and laser diffraction data was merged to form a complete particle size distribution. Folk and Ward (1957) geometric (modified) graphical (μm) measures were used for classifications. |
| *Chlorophyll a* | Sediment Chlorophyll samples were collected at 0-5cm depth intervals. These were freeze-dried and a known weight (~0.5g) of dried sediment extracted in 90% acetone using a modified method described by Tett et al (1987). The extracted pigment was measured using either spectrophotometry (DY008) (HMSO 1980) or fluorescence (DY021,DY030, and DY034) (Tett et al 1987). |
| *Porosity* | Porosity sediment samples were collected at 0-5cm depth intervals and frozen. They were subsequently defrosted, weighed, freeze dried and weighed again to get the dry:wet sediment weight ratio (Danielson and Sutherland 1986). |
| *Bulk Density* | 50ml syringe cores are taken and subsampled to 1cm sections. Wet bulk density is calculated as weight per volume of section. The sections are dried at 50^o^C and dry bulk density calculated as dry weight/volume of section. |
| *Permeability* | Calculated according to the equation of Engelund, 1953.  $K= \frac{\varepsilon^{2}d^{2}}{1000[1-\varepsilon]^{3}}$  Data are presented in the form of the coefficient of permeability, given by:  $k=K\frac{g}{\nu}$  Where, K is the specific permeability; g = acceleration due to gravity; v = kinematic viscosity, and d = sediment grain diameter. |
| *Organic Carbon and Nitrogen* | Samples were freeze dried, homogenized and subsampled to 100 mg into preweighed, acid-washed wide-mouth glass vials. Further sample preparation was carried out according to Hedges & Stern (1984): samples were subjected to subsequent aqueous acidification with HCl and redried. Subsamples of treated sediment (10–20 mg) were then weighed into tin cups for analysis following the methodology of Kirsten, 1979, using an elemental analyser (Flash EA 1112, Thermo-Finnigan) interfaced (ConFlo III Interface, Thermo-Finnigan) with a continuous flow isotope mass spectrometer (Finnigan MAT DeltaPlus, Thermo-Finnigan) calibrated against known quantities of urea. |
| *Oxygen Penetration Depth* | Sediment oxygen profiles were taken immediately from 10cm diameter cores, sub-sampled from NIOZ box cores using Unisense microelectrodes (Unisense OX500mm, Aarhus, Denmark) as described in Revsbech (1989). Calibration was based on 0% per cent oxygen readings recorded in ascorbate solution (0.2 M NaOH, 0.1 M ascorbic acid), and 100% per cent readings from aerated water overlying the core at ambient temperature and salinity for each core and replicate profile. The profiles were recorded continuously, using PICOLOG technology recorder software (Pico Technology). The oxygen penetration depth (OPD) was taken to be the depth at which oxygen saturation above 0% was last observed using a method adapted from Rabouille et al. (2003). |
| *Total Oxygen Consumption* | Full methods are presented elsewhere in this issue Hicks et al. (this issue), or can be found in Glud (2008). |
| *Zone of Mixing and Surface Roughness* | Both parameters were derived from Sediment Profile Images according to Teal et al, (2010) and Solan et al (2004). |

**Data availability**

Data are available through the British Oceanographic Data Centre (www.BODC.ac.uk)

| **Data Title** | **DOI** |
| --- | --- |
| Sediment classification data | 10.5285/47110529-757c-40b5-e053-6c86abc0eddc |
| *CTD Suspended Particulate Matter Concentrations* |  |
| DY008 | 10.5285/487b5547-e455-76b1-e053-6c86abc000a3 |
| DY021 | 10.5285/487b5547-e457-76b1-e053-6c86abc000a3 |
| DY030 | 10.5285/487b5547-e458-76b1-e053-6c86abc000a3 |
| DY034 | 10.5285/487b5547-e459-76b1-e053-6c86abc000a3 |
| *Benthic Landers and Smartbuoys* |  |
| CANDYFLOSS Smartbuoy | 10.14466/CefasDataHub.37 |
| Celtic Deep 2 Lander | 10.14466/CefasDataHub.38 |
| Celtic Deep 2 Smartbuoy | 10.14466/CefasDataHub.39 |
| East of Celtic Deep Lander | 10.14466/CefasDataHub.40 |
| East of Haig Fras Lander | 10.14466/CefasDataHub.41 |
| Nymph Bank Lander | 10.14466/CefasDataHub.42 |
|  |  |
| *Inorganic Nutrients* |  |
| Diffusive Flux Measurements | 10.5285/487b5547-e454-76b1-e053-6c86abc000a3 |
| Pore Water Nutrients | 10.5285/482da34c-86d8-5d39-e053-6c86abc044e3 |
| DY026 | 10.5285/2eb8d803-8823-1e6f-e053-6c86abc052a6 |
| JC105 | 10.5285/2eb8d803-8822-1e6f-e053-6c86abc052a6 |
|  |  |
| Oxygen and Ph. Profiles | 10.5285/47110529-757d-40b5-e053-6c86abc0eddc |
| Oxygen uptake rates | 10.5285/47110529-757b-40b5-e053-6c86abc0eddc |
|  |  |
| *Macrofaunal activity* |  |
| DY008 | 10.5285/48a674f7-e895-5f6c-e053-6c86abc0fd29 |
| DY021 | 10.5285/49214a48-665a-38af-e053-6c86abc0063a |
| DY030 | 10.5285/4931e463-60c4-3929-e053-6c86abc074c1 |
| DY034 | 10.5285/4931e463-60c5-3929-e053-6c86abc074c1 |
| Benthic nitrogen cycling – trawling and organic enrichment | 10.5285/46ecc183-c08d-2211-e053-6c86abc0d02c |

**References.**

Andrassy I. (1956) The determination of volume and weight of nematodes. Acta Zoologica (Hungarian Academy of Science) 2:1-1.

Heip C, Vincx M and Vranken G (1985) The Ecology of Marine Nematodes. Oceanography and Marine Biology 23:399-489.

Aquilina A, Homoky WB, Hawkes JA, Lyons TW and Mills RA (2014) Hydrothermal sediments are a source of water column Fe and Mn in the Bransfield Strait, Antarctica. Geochimica et Cosmochimica Acta 137:64-80.

Barnett PRO, Watson J and Connelly D (1984) A multiple corer for taking virtually undisturbed samples from shelf, bathyal and abyssal sediments Oceanologica Acta 7(4):399-408.

Danielson RE and Sutherland PL (1986) Porosity. In: Klute A (ed) Methods of Soil Analysis, Part 1, Physical and Mineralogical Methods. Agronomy Monograph No. 9. American Society of Agronomy, Soil Science Society of America, Madison, pp 443-461

Duplisea DE, Jennings S, Malcolm SJ, Parker R and Sivyer DB (2001) Modelling potential impacts of bottom trawl fisheries on soft sediment biogeochemistry in the North Sea. Geochemical Transactions 1(14):1-6.

Durden J, Bett B, Schoening T, Morris KJ, Nattkemper T and Ruhl H (2016) Comparison of image annotation data generated by multiple investigators for benthic ecology. Mar Ecol Prog Ser 552:61-70 doi:10.3354/meps11775.

Eigaard OR, Bastardie F, Breen M, Dinesen GE, Hintzen NT, Laffargue P, Mortensen LO, Nielsen JR, Nilsson HC, O’Neill FG, Polet H, Reid DG, Sala A, Sköld M, Smith C, Sørensen TK, Tully O, Zengin M and Rijnsdorp AD (2015) Estimating seabed pressure from demersal trawls, seines, and dredges based on gear design and dimensions. ICES Journal of Marine Science. doi: 10.1093/icesjms/fsv099.

Engelund F (1953) On the laminar and turbulent flow of ground water through homogeneous sand. Transaction, Danish Academy of Technical Sciences 3:105 pp.

Folk RL and Ward WC (1957) Brazos River bar: a study in the significance of grain size parameters. Journal of Sedimentary Petrology 27:3–26.

Germano JD, Rhoads DC, Valente RM, Carey DA and Solan M (2011) The use of sediment profile imaging (API) for environmental impact assessments and monitoring studies: lessons learned from the past four decades Oceanography and Marine Biology: An Annual Review 49:235-298.

Glud RN (2008) Oxygen dynamics of marine sediments. Marine Biology Research 4:243-289. doi: 10.1080/17451000801888726.

Hedges J & Stern J (1984) Carbon and nitrogen determinations of carbonate-containing solids. Limnol Oceanogr 28: 657–663.

Heip C, Vincx M and Vranken G (1985) The Ecology of Marine Nematodes. Oceanography and Marine Biology 23:399-489.

Hicks N, Ubbara G, Silburn B, Smith H, Kroger S, Parker R, Sivyer D, Kitidis V, Stahl H and Hatton A (2016) Oxygen dynamics in shelf seas sediments incorporating seasonal variability. Biogeochemistry. This Issue.

Higgins RP and Thiel H (1988) Introduction to the study of Meiofauna. Smithsonian Institution Press, London. 488 pp.

Homoky WB, John SG, Conway T and Mills RA (2013) Distinct iron isotopic signatures and supply from marine sediment dissolution. Nature Communications 4: doi: 10.1038/ncomms3143.

Homoky WB, Severmann S, McManus J, Berelson WM, Riedel TE, Mills RA and Statham PJ (2012) Dissolved oxygen and suspended particles regulate the benthic flux of iron from continental margins. *Marine Chemistry* 134-135:59-70

HMSO. (1980). The determination of chlorophyll a in aquatic environments 1980. In: Methods for the estimation of waters and associated materials. HMSO 1980, ISBN 0 11 751674 0.

Hydes DJ, Aoyama M, Aminot A, Bakker K, Becker S, Coverly S, Daniel A, Dickson AG, Grosso O, Kerouel R, van Ooijen J, Sato K, Tanhua T, Woodward EMS and Zhang JZ (2010) The GO-SHIP Repeat Hydrography Manual: a collection of expert reports and guidelines; IOCCP report No.14, ICPO publication series No. 134, version 1.

Jennings S, Lancaster J, Woolmer A and Cotter J (1999) Distribution, diversity and abundance of epibenthic fauna in the North Sea, *Journal of the Marine Biological Association of the United Kingdom* 79(3):385–399.

Kallmeyer J, Pockalny R, Adhikari RR, Smith DC, and D’Hondt S (2012) Global distribution of microbial abundance and biomass in subseafloor sediment. Proceedings of the National Academy of Sciences 109:16213-16216.

Klar J Statham PJ, Homoky WB, Woodward EMS, Chever F, Lichtschlag A, Harris E, Silburn B (2016) The seasonal occurrence and stability of dissolved and soluble Fe(II) generated in an oxic shelf environment, Biogeochemistry, This issue.

Kristen WJ (1979) Automatic methods for the simultaneous determination of Carbon, Hydrogen, Nitrogen and Sulfur, and for Sulfur alone in organic and inorganic materials. Analytical Chemistry 51(8):1173-1179

Natale F, Gibin M, Alessandrini A, Vespe M and Paulrudm A (2015) Mapping fishing effort through AIS data. PLoS ONE 10(6):e0130746.

NMBAQC's Best Practice Guidance. Particle Size Analysis (PSA) for Supporting 634 Biological Analysis. National Marine Biological AQC Coordinating Committee. 635-636

Manini E and Danovaro R (2006) Synoptic determination of living/dead and active/dormant bacterial fractions in marine sediments. FEMS microbiology ecology 55:416-423.

Mason CE (2011) NMBAQC's Best Practice Guidance. Particle Size Analysis (PSA) for Supporting Biological Analysis. National Marine Biological AQC Coordinating Committee.

McCauley DJ, Woods P, Sullivan B, Bergman B, Jablonicky C, Roan A, Hirshfield M, Boerder K and Worm B (2016) Ending hide and seek at sea. Science 351:1148-1150.

Morris K, Bett B, Durden J, Huvenne V, Milligan R, Jones D, McPhail S, Robert K, Bailey D & Ruhl H (2014) A new method for ecological surveying of the abyss using autonomous underwater vehicle photography. Limnology and Oceanography: Methods 12:795-809.

Rabouille C, Denis L, Dedieu K, Stora G, Lansard B & Grenz C (2003) Oxygen demand in coastal marine sediments: comparing in situ microelectrodes and laboratory core incubations. J Exp Mar Biol Ecol 285–286: 49–69.

Revsbech N (1989) An oxygen microsensor with a guard cathode. Limnol Oceanogr 34: 474–478.

Rhoads DC and Cande S (1971) Sediment profile camera for in situ study of organism-sediment relations. Limnology and Oceanography 16(1):110–114. <http://doi.org/10.4319/lo.1971.16.1.0110>.

Ribas-Ribas M, Rérolle VMC, Bakker DCE, Kitidis V, Lee GA, Brown I, Achterberg EP, Hardman-Mountford NJ and Tyrrell T (2014) Intercomparison of carbonate chemistry measurements on a cruise in northwestern European shelf seas. Biogeosciences 11 4339-4355, doi:10.5194/bg-11-4339-2014.

Sciberras M, Parker R, Powell C, Robertson C, Krӧger S, Bolam S and Hiddink JG (2016) Impacts of bottom fishing on the sediment infaunal community and biogeochemistry of cohesive and non-cohesive sediments. Limnology and Oceanography 10.1002/lno.10354.

Seeberg-Elverfeldt J, Schlüter M, Feseker T and Kölling M (2005) Rhizon sampling of porewaters near the sediment-water interface of aquatic systems. Limnology and Oceanography: Methods 3(8):361-371.

Solan M, Wigham B, Hudson I, Kennedy R, Coulon C, Norling K, Nilsson H and Rosenberg R (2004) In situ quantification of bioturbation using time-lapse fluorescent sediment profile imaging (f-SPI), luminophore tracers and model simulation. Marine Ecology Series 271:1-12.

Somerfield P and Warwick R (1996) Meiofauna in marine pollution monitoring programmes: a laboratory manual. MAFF Directorate of Fisheries Research Technical Series 71 pp.

Stookey LL (1970) Ferrozine - a new spectrophotometric reagent for iron. Analytical Chemistry 42(7):779-781.

Tait K, Stahl H, Taylor P and Widdicombe S (2015) Rapid response of the active microbial community to CO_2_ exposure from a controlled sub-seabed CO_2_ leak in Ardmucknish Bay (Oban, Scotland). International Journal of Greenhouse Gas Control 38:171-181.

Teal LR, Parker ER and Solan M (2010) Sediment mixed layer as a proxy for benthic ecosystem process and function. Marine Ecology Progress Series 414:27-40.

Tett P (1987) Plankton. In: Biological surveys of estuaries and coasts. Estuarine and brackish water sciences association handbook (Eds. Baker JM and Wolff WJ). Cambridge University Press, Cambridge, U.K. 280-341.

Trimmer M, Nedwell DB, Sivyer DB and Malcolm SJ (2000) Seasonal benthic organic matter mineralisation measured by oxygen uptake and denitrification along an inner and outer transect of the River Thames estuary U.K. Marine Ecology Progress Series 197:103-119.

Trimmer M, Petersen J, Sivyer DB, Mills C, Young E and Parker ER (2005) Impact of long-term benthic trawl disturbance on sediment sorting and biogeochemistry in the southern North Sea. Marine Ecology Progress Series 298:79-94.

Waterbury RD, Wensheng Y and Byrne RH (1997) Long pathlength absorbance spectroscopy: trace analysis of Fe(II) using a 4.5 m liquid core waveguide. Analytica Chimica Acta 357(1–2):99-102.

Welshmeyer NA (1994) Fluorometric analysis of chlorophyll a in the presence of chlorophyll b and phaeopigments. Limnology and Oceanography 39:1985-1992.

Weston K, Fernand L, Nicholls J, Marca-Bell A, Mills D, Sivyer D and Trimmer M (2008) Sedimentary and water column processes in the Oyster Grounds: A potentially hypoxic region of the North Sea. Marine Environmental Research 65(3):235-249.

Woodward EMS and Rees AP (2001) Nutrient distributions in an anticyclonic eddy in the North East Atlantic Ocean, with reference to nanomolar ammonium concentrations. Deep Sea Research II 48(4-5):775-794.
