# Supplementary material for: An approach for the identification of exemplar sites for scaling up targeted field observations of benthic biogeochemistry in heterogeneous environments
Source: Biogeochemistry. 2017 Aug 1;135(1):1–34. doi: 10.1007/s10533-017-0366-1 (PMC6961521; doi:10.1007/s10533-017-0366-1)
Supplement: Supplementary file 2 — Long Term Observation deployment positions and operation timescales (DOCX 78 kb) [file 10533_2017_366_MOESM2_ESM.docx]

Online Resource 2: Long Term Observation deployment positions.

| **Site** | **Latitude** | **Longitude** | **Start Date** | **End Date** | **Platform** |
| --- | --- | --- | --- | --- | --- |
| **Celtic Deep** | 51.250 | -6.082 | 05 April 2009 | 30 April 2012  00:00 | SmartBuoy |
| **Celtic Deep 2** | 51.138 | -6.562 | 15 July 2012 | 19 August 2015  00:00 | SmartBuoy |
| **Candyfloss** | 49.402 | -8.604 | 27 March 2014 | 24 August 2015  00:00 | SmartBuoy |
| **Nymph Bank** | 51.043 | -6.600 | 31 January 2014 | 27 August 2014  00:00 | Lander |
| **East of Haig Fras** | 50.569 | -7.022 | 31 January 2014 | 30 August 2015  20:00 | Lander |
| **East of Celtic Deep** | 21.126 | -6.177 | 18 March 2014 | 21 October 2014  00:00 | Lander |
| **Celtic Deep 2 Lander** | 21.136 | -6.568 | 20 October 2014 | 30 August 2015  00:00 | Lander |
| **M5 Wave Buoy** | 51.690 | -6.704 | 18 October 2004 | 31 December 2015 | Met Buoy |
