# Supplementary material for: An approach for the identification of exemplar sites for scaling up targeted field observations of benthic biogeochemistry in heterogeneous environments
Source: Biogeochemistry. 2017 Aug 1;135(1):1–34. doi: 10.1007/s10533-017-0366-1 (PMC6961521; doi:10.1007/s10533-017-0366-1)
Supplement: Supplementary file 3 — Spatial survey sediment characteristics, organised by % Fines < 63 μm (DOCX 174 kb) [file 10533_2017_366_MOESM3_ESM.docx]

Online Resource 3: Spatial survey sediment characteristics, organised by % Fines < 63μm.

| **Station ID** | **Textural Group** | **Mean** | **Sorting** | **Skewness** | **Kurtosis** | **D_50_ (μm)** | **% Fines** |
| --- | --- | --- | --- | --- | --- | --- | --- |
| **Spatial 48** | Gravelly Sand | 873.3 | 2.986 | 0.404 | 1.130 | 671.9 | 1.73 |
| **Spatial 45** | Slightly Gravelly Sand | 386.1 | 1.591 | 0.081 | 1.183 | 373.1 | 2.55 |
| **Spatial 16** | Slightly Gravelly Sand | 368.4 | 1.833 | -0.007 | 1.325 | 355.5 | 4.02 |
| **Spatial 14** | Slightly Gravelly Sand | 412.9 | 1.788 | -0.090 | 1.280 | 402.2 | 4.09 |
| **Spatial 53** | Slightly Gravelly Sand | 483.1 | 2.006 | -0.099 | 1.457 | 478.1 | 4.16 |
| **Spatial 42** | Slightly Gravelly Sand | 409.2 | 1.836 | -0.109 | 1.163 | 405.9 | 4.28 |
| **Spatial 49** | Slightly Gravelly Sand | 334.7 | 2.049 | -0.026 | 1.375 | 318.9 | 4.82 |
| **Spatial 12** | Slightly Gravelly Sand | 348.2 | 2.106 | -0.165 | 2.116 | 338.1 | 5.45 |
| **Spatial 13** | Slightly Gravelly Sand | 398.9 | 2.206 | -0.256 | 2.280 | 394.2 | 5.92 |
| **Spatial 44** | Slightly Gravelly Sand | 433.5 | 2.494 | -0.309 | 2.583 | 430.3 | 6.65 |
| **Spatial 4** | Slightly Gravelly Sand | 407.7 | 2.485 | -0.285 | 2.404 | 403.6 | 6.75 |
| **Spatial 52** | Gravelly Muddy Sand | 778.8 | 4.142 | -0.376 | 1.963 | 896.2 | 8.80 |
| **Spatial 11** | Slightly Gravelly Muddy Sand | 320.7 | 2.814 | -0.360 | 3.370 | 332.6 | 10.49 |
| **Spatial 43** | Gravelly Muddy Sand | 1076.7 | 11.427 | 0.213 | 1.227 | 476.3 | 12.04 |
| **Spatial 37** | Slightly Gravelly Muddy Sand | 299.6 | 3.813 | -0.614 | 2.531 | 436.6 | 14.20 |
| **Spatial 5** | Slightly Gravelly Muddy Sand | 284.8 | 4.273 | -0.459 | 2.130 | 367.2 | 14.96 |
| **Spatial 35** | Slightly Gravelly Muddy Sand | 292.2 | 4.748 | -0.465 | 1.895 | 405.8 | 15.28 |
| **Spatial 21** | Slightly Gravelly Muddy Sand | 131.1 | 3.515 | -0.085 | 1.751 | 118.6 | 18.61 |
| **Spatial 55** | Slightly Gravelly Muddy Sand | 104.5 | 4.234 | -0.280 | 2.005 | 117.9 | 21.50 |
| **Spatial 34** | Slightly Gravelly Muddy Sand | 113.1 | 4.646 | -0.380 | 1.609 | 145.4 | 22.41 |
| **Spatial 51** | Slightly Gravelly Muddy Sand | 116.6 | 5.480 | -0.402 | 1.443 | 166.7 | 23.48 |
| **Spatial 36** | Slightly Gravelly Muddy Sand | 145.8 | 6.735 | -0.643 | 1.109 | 329.9 | 25.18 |
| **Spatial 6** | Slightly Gravelly Muddy Sand | 128.5 | 6.424 | -0.663 | 1.136 | 303.3 | 25.67 |
| **Spatial 15** | Slightly Gravelly Muddy Sand | 141.4 | 7.362 | -0.574 | 1.106 | 306.6 | 25.94 |
| **Spatial 10** | Slightly Gravelly Muddy Sand | 112.1 | 7.054 | -0.619 | 1.241 | 263.9 | 25.98 |
| **Spatial 22** | Slightly Gravelly Muddy Sand | 94.4 | 6.914 | -0.375 | 1.282 | 145.9 | 27.05 |
| **Spatial 50** | Slightly Gravelly Muddy Sand | 94.9 | 6.751 | -0.540 | 1.238 | 190.8 | 27.70 |
| **Spatial 31** | Slightly Gravelly Muddy Sand | 79.6 | 5.580 | -0.381 | 1.539 | 115.7 | 28.41 |
| **Spatial 32** | Slightly Gravelly Muddy Sand | 78.5 | 6.992 | -0.345 | 1.031 | 120.4 | 31.45 |
| **Spatial 29** | Slightly Gravelly Muddy Sand | 71.5 | 6.719 | -0.384 | 1.235 | 112.9 | 31.71 |
| **Spatial 54** | Muddy Sand | 68.1 | 7.038 | -0.359 | 1.215 | 104.4 | 31.91 |
| **Spatial 33** | Slightly Gravelly Muddy Sand | 58.1 | 6.179 | -0.430 | 1.503 | 98.1 | 32.96 |
| **Spatial 25** | Slightly Gravelly Muddy Sand | 70.8 | 7.637 | -0.316 | 1.091 | 104.3 | 33.88 |
| **Spatial 30** | Slightly Gravelly Muddy Sand | 64.8 | 6.926 | -0.398 | 1.118 | 105.3 | 34.03 |
| **Spatial 18** | Slightly Gravelly Muddy Sand | 60.5 | 7.221 | -0.378 | 1.108 | 96.0 | 35.98 |
| **Spatial 40** | Slightly Gravelly Muddy Sand | 63.1 | 6.994 | -0.272 | 1.300 | 86.7 | 37.05 |
| **Spatial 24** | Slightly Gravelly Muddy Sand | 55.1 | 6.799 | -0.412 | 1.140 | 91.8 | 37.06 |
| **Spatial 57** | Slightly Gravelly Muddy Sand | 56.8 | 6.867 | -0.352 | 1.184 | 88.2 | 37.54 |
| **Spatial 19** | Slightly Gravelly Muddy Sand | 58.6 | 7.235 | -0.410 | 0.945 | 99.5 | 37.85 |
| **Spatial 28** | Slightly Gravelly Muddy Sand | 49.5 | 6.398 | -0.450 | 1.070 | 87.2 | 38.71 |
| **Spatial 7** | Slightly Gravelly Muddy Sand | 68.5 | 6.785 | -0.416 | 0.878 | 115.2 | 38.93 |
| **Spatial 23** | Slightly Gravelly Muddy Sand | 49.3 | 6.288 | -0.436 | 1.066 | 83.9 | 39.64 |
| **Spatial 39** | Slightly Gravelly Muddy Sand | 44.7 | 5.298 | -0.471 | 1.367 | 75.4 | 41.04 |
| **Spatial 26** | Slightly Gravelly Muddy Sand | 50.9 | 6.953 | -0.364 | 0.953 | 80.9 | 42.59 |
| **Spatial 27** | Slightly Gravelly Muddy Sand | 40.9 | 7.557 | -0.338 | 1.026 | 67.9 | 47.44 |
| **Spatial 2** | Slightly Gravelly Sandy Mud | 41.1 | 8.749 | -0.171 | 0.804 | 49.0 | 53.25 |
| **Spatial 38** | Slightly Gravelly Sandy Mud | 27.5 | 6.606 | -0.276 | 1.091 | 40.3 | 60.84 |
| **Spatial 9** | Sandy Mud | 12.8 | 5.505 | -0.098 | 1.077 | 13.3 | 83.70 |
| **Spatial 8** | Sandy Mud | 13.1 | 5.020 | -0.169 | 0.976 | 14.1 | 84.54 |
| **Spatial 1** | Sandy Mud | 12.8 | 5.142 | -0.128 | 1.035 | 13.4 | 84.61 |
| **Spatial 3** | Sandy Mud | 12.3 | 4.954 | -0.144 | 1.030 | 12.9 | 86.61 |
