# Supplementary figures and images for: An approach for the identification of exemplar sites for scaling up targeted field observations of benthic biogeochemistry in heterogeneous environments
Source: Biogeochemistry. 2017 Aug 1;135(1):1–34. doi: 10.1007/s10533-017-0366-1 (PMC6961521; doi:10.1007/s10533-017-0366-1)

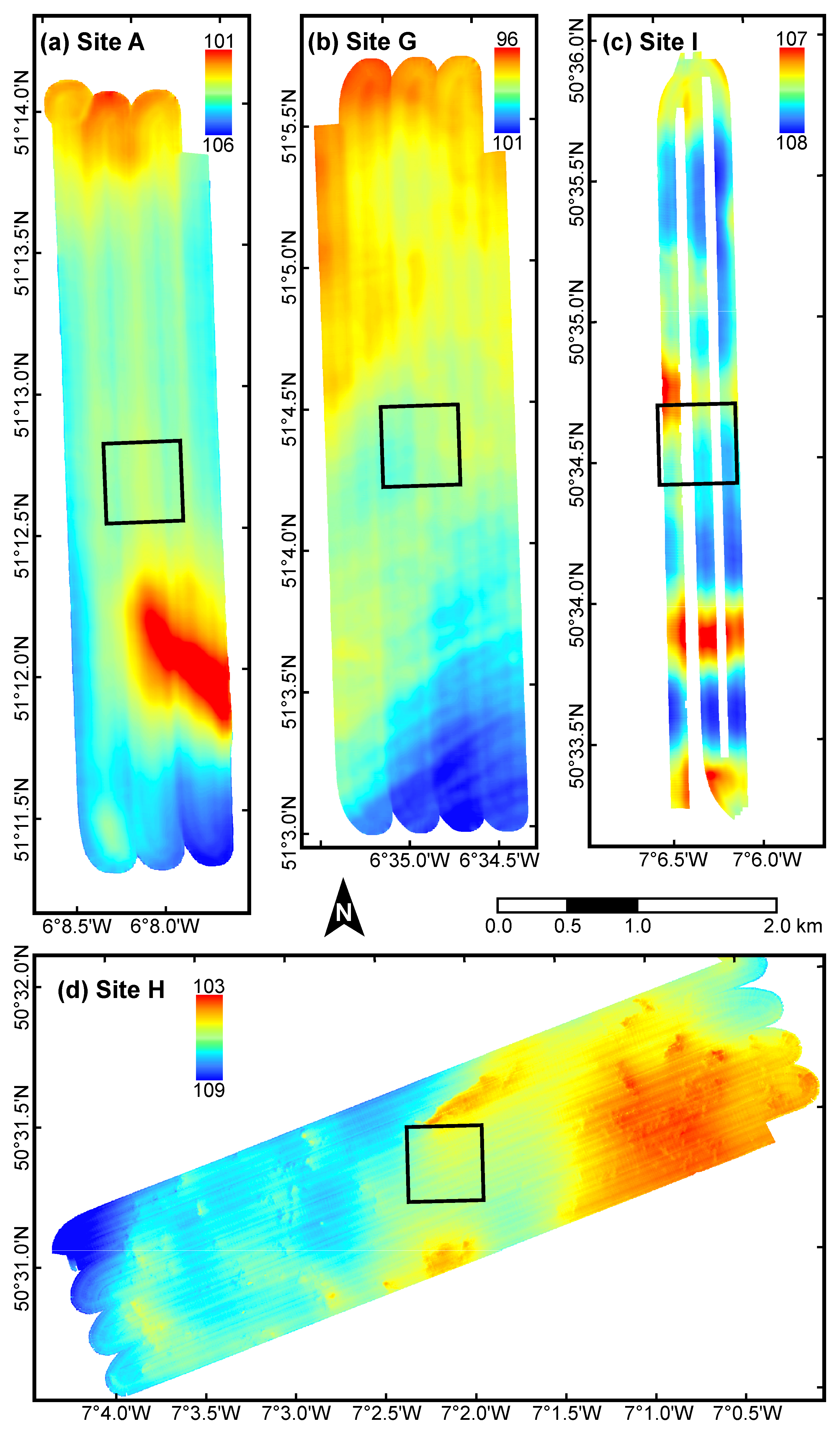

Supplement: Supplementary file 4 — Bathymetric maps generated from Autosub3 (a) site A, (b) site G, (d) site I and Autosub6000 (d) site H multibeam data (smoothed at 50 m horizontal scale). Water depth ranged from 101-106 m at Site A, with the study box having a general depth of 103 m; 96-101 m at Site G, study box general depth of 98 m; 106-107 m at site I, study box general depth of 107 m; 103-109 m at Site H, study box general depth of 105 m (TIFF 4187 kb) [file 10533_2017_366_MOESM4_ESM.tif]

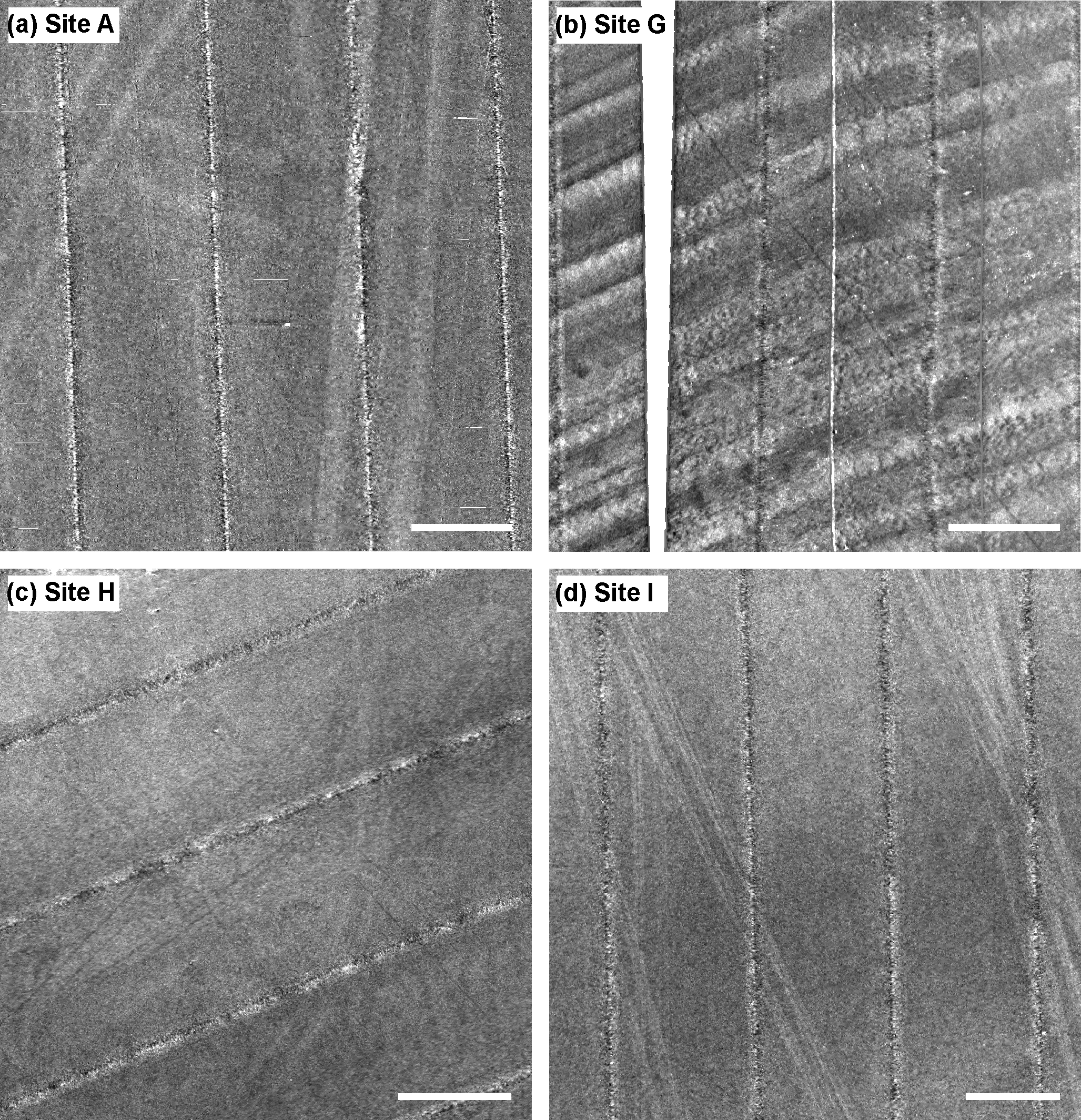

Supplement: Supplementary file 5 — Sidescan of the 4 study sites: (a) Site A, (b) Site G, (c) Site H and (d) Site I. All scale bars represent 100 m. The parallel white lines are the nadir and represent lines with no data. Note that at Site G, the white vertical band represents an area where no data were collected. The presence of repeating backscatter ‘stripes’ at Site G is clear and appear to be matched by bathymetric variations suggestive of sedimentary bedforms. Presumed “trawl marks” (seabed scars resulting from commercial bottom trawling operations) are particularly notable at Site I, but also present at sites A, G and H (TIFF 5871 kb) [file 10533_2017_366_MOESM5_ESM.tif]

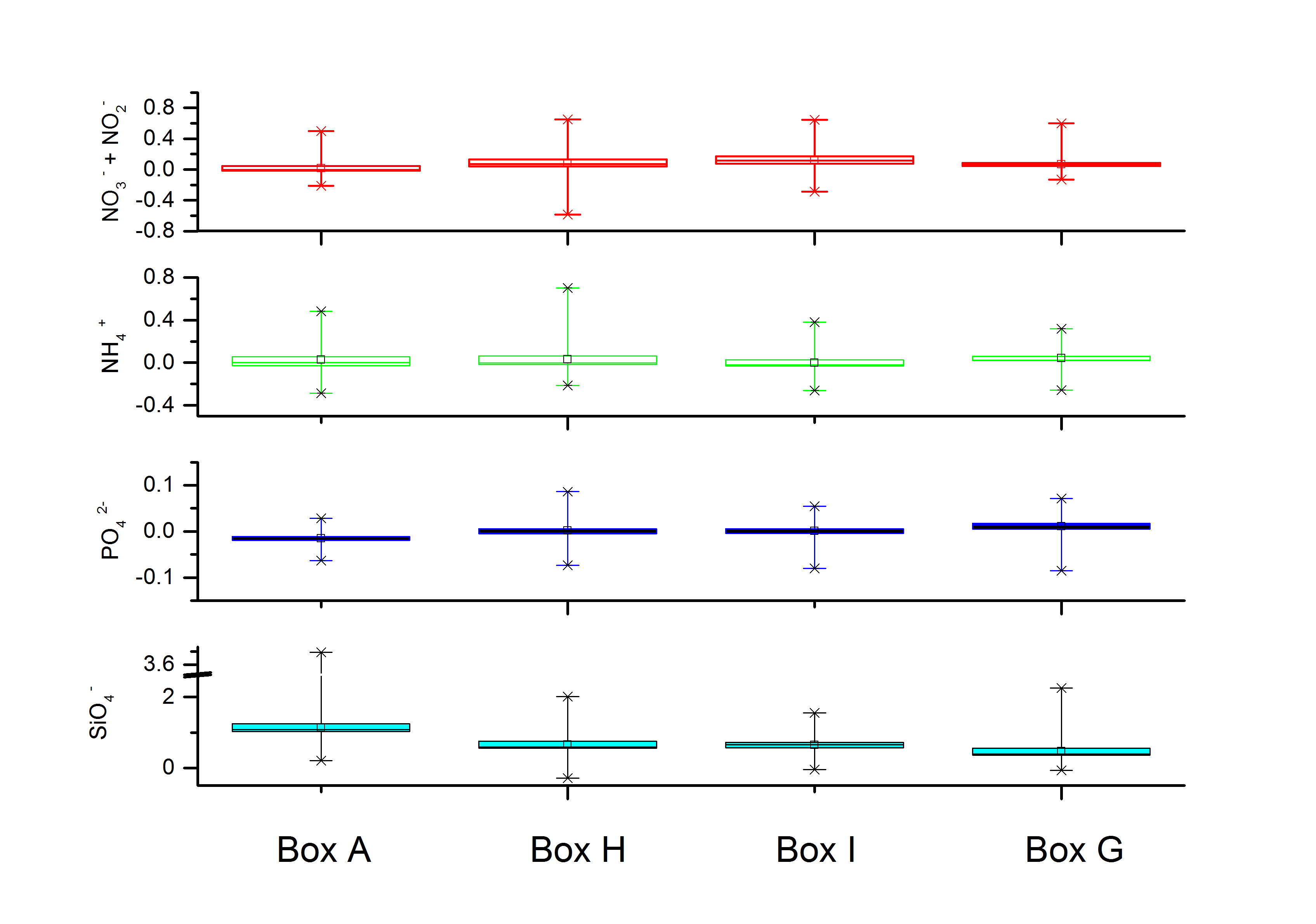

Supplement: Supplementary file 6 — Mean Nutrient Fluxes (mmol.m−2.d−1) averaged over all seasons: Boxes represent mean and SE, with max and min whiskers (DOCX 783 kb) [file 10533_2017_366_MOESM6_ESM.docx]

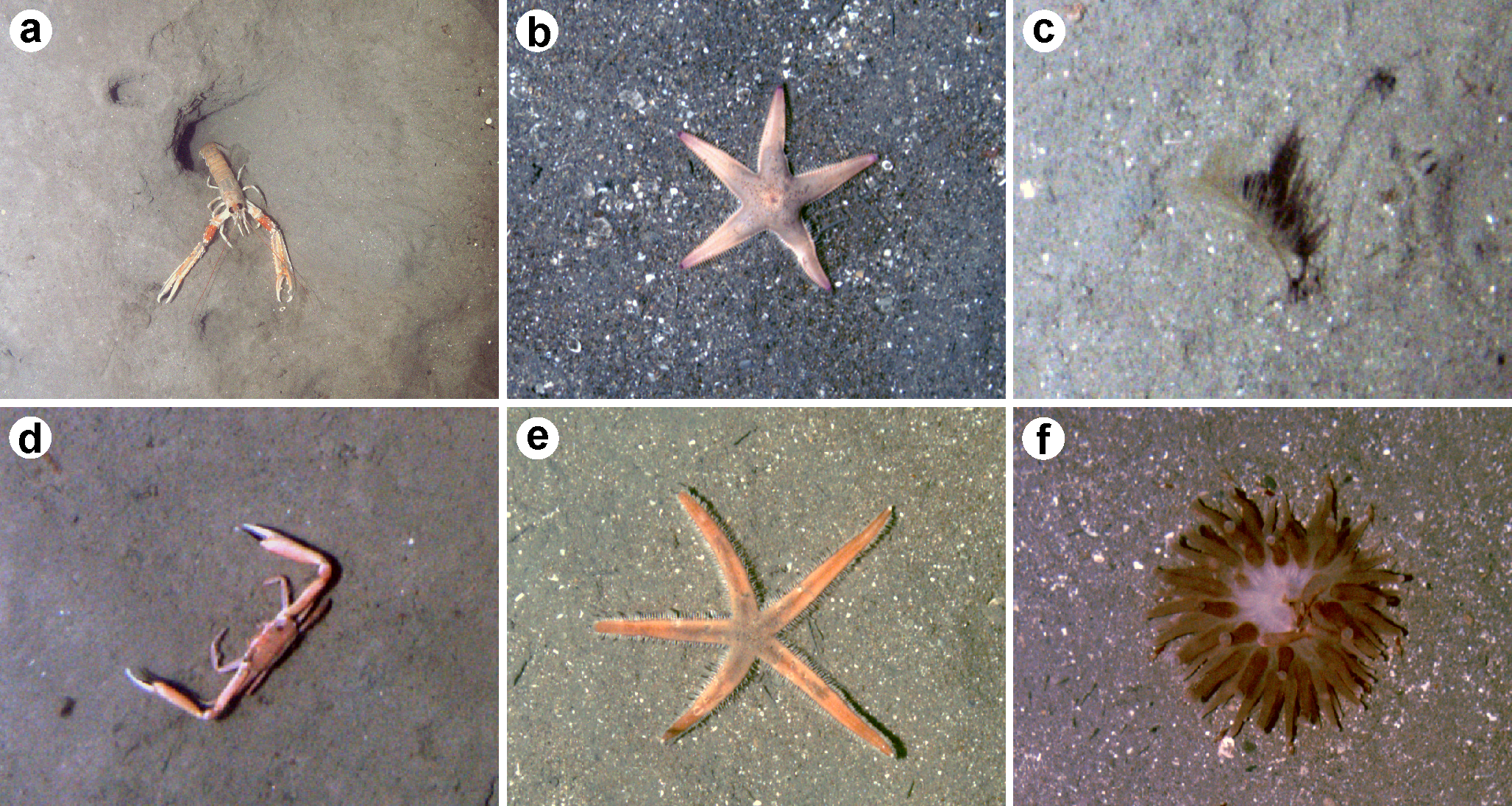

Supplement: Supplementary file 7 — Example of density dominant fauna across sites G, H and I. Taxa were determined to the lowest taxonomic level whenever possible (otherwise a morphotype was assigned). Arthropoda (a) Nephrops norvegicus and (d) Goneplax rhomboides; Echinodermata Asteroidea (b) Astropecten irregularis, (f) Luidia sarsii; and, Cnidaria (c) Cnidaria spp. type 01, (f) Bolocera spp. type 01 (TIFF 4930 kb) [file 10533_2017_366_MOESM7_ESM.tif]
