# Supplementary material for: An approach for the identification of exemplar sites for scaling up targeted field observations of benthic biogeochemistry in heterogeneous environments
Source: Biogeochemistry. 2017 Aug 1;135(1):1–34. doi: 10.1007/s10533-017-0366-1 (PMC6961521; doi:10.1007/s10533-017-0366-1)
Supplement: Supplementary file 8 — Site specific species abundance (DOCX 125 kb) [file 10533_2017_366_MOESM8_ESM.docx]

**Online Resource 8:** Site specific species abundance

The epifauna at site A was dominated by the bivalve *Nucula sulcata*, the commercially important *Nephrops norvegicus* or “scampi/Dublin Bay Prawn”, several species of shrimp (*Crangon allmanni, Dichelopandalus bonnieri, Alpheus glaber, Processa nouveli holthuisi*), the angular crab (*Goneplax rhomboides*) and the predatory gastropod *Polinices fuscus*. At site I, many of the large bioturbatory species that were highly abundant at site A were also present in this slightly sandier environment (e.g. *N. norvegicus, G. rhomboides* and *N. sulcata*) although at much lower abundances. Another important bioturbator, the burrowing sandstar *Astropecten irregularis* was also present but at lower abundance than seen at the sandier site H, as were the shrimps *Processa canaliculata* and *Philoceras bispinosus*. Many species that were common to sites A, I and H showed their highest abundances at site I, these included *C. allmanni, P. nouveli holthuisi, A. glaber, Pontophilus spinosus, Ampelisca macrocephala* and *Ophiura affinis.*

Several species showed their highest abundances in the sediments of site H. These included *P. canaliculata, P. bispinosus* and *A. irregularis*. Several species were found in high abundance at this site whilst being rare at all other sites; e.g. the terrebellid worm *Amphicteis gunneri*, the infaunal gastropod *Turritella communis* and the bivalve *Phaxas pellucidus*. Unlike these species, *P. nouveli holthuisi* showed similarly high abundances in sites A, I and H. The shrimp *C. allmanni* was abundant at sites A and I and, although it was still found at site H, its abundance was reducing as the sediment type became coarser The opposite was true for the hermit crab *Anapagurus laevis* which appeared to become more abundant in sandier sediments.

The most abundant epifaunal species in the coarse sand sediments found at site G was the tube building polychaete *Hyalinoecia tubicola*. This species was almost exclusively found at site G, as were the pelican’s foot shell *Aporrhais pespelecani* and the nereid worm *Neanthes fucata,* which is a commensal of *Paguridae*. site G also supported several species of hermit crabs (*Pagurus bernhardus, Anapagurus. laevis, Pagurus prideaux* and *Pagurus alatus*) all of which were most abundant in sandy environments. The crabs *Ebalia granulosa* and *Liocarcinus depurator,* as well as the brittlestar *Ophiura sarsi,* were also more abundant at sandier sites showing high numbers at both sites G and H. Although the shrimps *C. allmanni* and *P. bispinosus* were still abundant at site G, it would seem that these species actually favoured sediments with a higher percentage of fines.

The most numerically dominant macro-infaunal species at site A was the capitellid polychaete *Mediomastus fragilis* with more than twice as many individuals as the next most abundant species, the bivalve *Abra nitida* and the polychaetes *Minuspio cf. multibranchiata*, *Magelona minuta*, *Abyssoninoe hibernica* and *Praxillella affinis.* Other less abundant taxa were *Nemertea*, another polychaete *Prionospio fallax* and the cumacean *Leucon nasica*. Site I was even more heavily dominated by its most abundant species, the polychaete *Magelona minuta*, being 8 times more abundant than any other taxa. These other taxa included *Nemertea*, the polychaetes *Ampharete falcata*, *Mediomastus fragilis*, *Aricidea suecica* and *Galathowenia oculata* as well as the bivalve *Corbula gibba*. Site H was similar to site I in terms of dominant species and was also heavily dominated by *M. minuta*. However at site H this species was only 5 times more abundant than the next most abundant taxa, which were juvenile brittlestars *Amphiuridae*, *Nemertea*, the polychaetes *A. falcata* and *A. suecica* and the amphipod *Urothoe elegans*. This site also contained significant numbers of the urchin *Echinocyamus pusillus* indicating the sandier nature of this site compared to site I. Site G was numerically dominated by echinoderms with juvenile *Amphiuridae* and *E. pusillus* being 3 times more abundant than any other taxa. The next most abundant were *Unciola planipes*, *Spiophanes kroyeri* and Nemertea.

Meiofaunal nematodes, copepods, bivalves and polychaetes were present at all sites, but ostracods and kinorhynchs only occurred at A and I, while rotifers were only recovered from G and H sediments. Nematode and copepod relative abundance differed significantly between sites (p = 0.032, p = 0.002, respectively), and is likely a useful indicator of sedimentary differences between sites. Phyla richness, – although a very rough measure of meiofauna diversity, – was significantly different between sites H and I (p = 0.012). However, we expect such differences between sites to be greater when considering nematode genera/species data.
